# Phage-Plasmids Spread Antibiotic Resistance Genes through Infection and Lysogenic Conversion

**DOI:** 10.1128/mbio.01851-22

**Published:** 2022-09-26

**Authors:** Eugen Pfeifer, Rémy A. Bonnin, Eduardo P. C. Rocha

**Affiliations:** a Institut Pasteur, Université Paris Cité, CNRS UMR3525, Microbial Evolutionary Genomics, Paris, France; b Team “Resist” UMR1184 “Immunology of Viral, Auto-Immune, Hematological and Bacterial diseases (IMVA-HB),” INSERM, Université Paris-Saclay, CEA, LabEx LERMIT, Faculty of Medicine, Associated French National Reference Center for Antibiotic Resistance: Carbapenemase-Producing Enterobacteriaceae, Le Kremlin-Bicêtre, France; Universidade de Sao Paulo

**Keywords:** antibiotic resistance, bacteriophages, integrons, phage genomics, plasmids, prophage induction

## Abstract

Antibiotic resistance is rapidly spreading via the horizontal transfer of resistance genes in mobile genetic elements. While plasmids are key drivers of this process, few integrative phages encode antibiotic resistance genes. Here, we find that phage-plasmids, elements that are both phages and plasmids, often carry antibiotic resistance genes. We found 60 phage-plasmids with 184 antibiotic resistance genes, providing resistance for broad-spectrum-cephalosporins, carbapenems, aminoglycosides, fluoroquinolones, and colistin. These genes are in a few hot spots, seem to have been cotranslocated with transposable elements, and are often in class I integrons, which had not been previously found in phages. We tried to induce six phage-plasmids with resistance genes (including four with resistance integrons) and succeeded in five cases. Other phage-plasmids and integrative prophages were coinduced in these experiments. As a proof of concept, we focused on a P1-like element encoding an extended spectrum β-lactamase, *bla*_CTX-M-55_. After induction, we confirmed that it is capable of infecting and converting four other E. coli strains. Its reinduction led to the further conversion of a sensitive strain, confirming that it is a fully functional phage. This study shows that phage-plasmids carry a large diversity of clinically relevant antibiotic resistance genes that they can transfer across bacteria. As plasmids, these elements seem plastic and capable of acquiring genes from other plasmids. As phages, they may provide novel paths of transfer for resistance genes because they can infect bacteria that are distant in time and space from the original host. As a matter of alarm, they may also mediate transfer to other types of phages.

## INTRODUCTION

Antimicrobial resistance (AMR) is fast disseminating among human-associated bacteria and has been classified as a major challenge to Global Health (1). Enterobacterales are identified as the most critical group (2) against which new drugs need to be developed. Resistance is the result of one of multiple mechanisms: limiting drug uptake; target modification; active drug efflux, and drug inactivation. The latter includes the extended spectrum β-lactamases (ESBLs) that allow Enterobacterales to become resistant against most β-lactams (such as penicillins or broad-spectrum cephalosporins). Although ESBLs do not directly provide resistance to carbapenems (last-resort antibiotics within β-lactams), the wide and improper use of carbapenems, especially as a first-line treatment, has promoted the emergence of carbapenem-resistant Enterobacterales (CRE) strains that are commonly found to be resistant to other antibiotic classes (3). While low-level resistance to β-lactams can be provided by many mechanisms, such as qualitative or quantitative modifications of porins, high resistance is usually associated with the acquisition of genes encoding ESBLs or carbapenemases via horizontal gene transfer (4). The most important and clinically relevant carbapenemases identified in Enterobacterales belong to class A (KPC-like enzymes), class B (NDM-, VIM- and IMP-like enzymes), or class D (OXA-48-like enzymes) type β-lactamases (5).

Plasmids are key drivers of the transmission of antibiotic resistance genes (ARGs) between bacteria, and this transmission usually occurs via conjugation (6, 7). The transfer is also facilitated by the presence of mobile genetic elements (MGEs) that translocate genetic information between replicons (8). Notably, ARGs are often flanked by transposable elements that facilitate their translocation between plasmids or between a plasmid and the chromosomes (9). Integrons can also facilitate the translocation of ARG cassettes (10). Mobile integrons are usually associated with plasmids and/or transposons and consist of one integrase (here, of the type IntI1) and a small array of gene cassettes that are flanked by recombination sites. Integrons can acquire new gene cassettes from other integrons and can shuffle existing ones (11). A large fraction of the cassettes of mobile integrons consists of ARGs (10). The cotransfer of multiple ARGs in an integron facilitates the emergence of multidrug resistance strains.

Temperate bacteriophages (phages) can mobilize genes via different types of transduction processes (generalized, specialized, and lateral) (12) or can introduce new genes by lysogenic conversion (13). Generalized transduction relies on the erroneous packaging of nonphage DNA by specific types of phages and tends to occur at low frequencies (14), whereas lateral and specialized transduction require proximity between the transferring genes and the phage (12). All of these processes have been shown to result in the transfer of ARGs in the lab, but there is extensive controversy regarding the extent and pertinence of this process in natural environments (15–19). In contrast, lysogeny is common in nature (20–22). In this case, the phage remains mostly silent in the cell (as a prophage), but its accessory genes can be expressed and change the phenotype of the host. Many toxins with key impacts on the virulence of bacterial pathogens are present and are expressed from prophages (13). However, few phages encode bona fide ARGs (16). To the best of our knowledge, no natural phage with ARGs has been shown to be fully functional, that is, to be capable of lysing the original host cell, infecting another cell, and then repeating the cycle to infect a third cell, thereby providing antibiotic resistance by lysogenic conversion.

While most prophages integrate the chromosome, some remain in cells as phage-plasmids (P-Ps). These are temperate phages that transfer horizontally (infect) as viruses but remain and replicate within cells as plasmids. In a previous work, we found P-Ps to be numerous, widespread, and organized into different groups (23). A few of these groups are frequent in Enterobacteria and in other important nosocomial pathogens. For example, P1-like P-Ps are frequent in Escherichia coli, SSU5-like and N15-like elements are frequent in Klebsiella pneumoniae, and AB-like P-Ps are frequent in Acinetobacter baumannii. P-Ps tend to be larger than prophages integrated into a chromosome. They have loci that are plastic and contain genes typical of plasmids and, more conserved loci that encode phage-related genes (23). Some of the P-Ps, notably the P1-like, can also be efficient transducers (24). The double nature of P-Ps, being a plasmid and a phage, leads us to think that they might contribute more, especially via lysogenic conversion, to the spread of ARGs than do other phages. Furthermore, several reports have identified elements resembling P-Ps carrying ARGs. For example, P1-like elements were identified as encoding an *mcr-1* gene that conferred resistance to colistin in K. pneumoniae and as ESBLs in Salmonella spp. and E. coli, but their induction and transmission could not be confirmed (25–27). Recently, a P1-like element with several predicted ARGs was demonstrated to be able to lysogenize one commensal E. coli strain and provide resistance to streptomycin (28). This shows that P-Ps can carry and transfer ARGs, although the viability of the full phage life cycle (infection and reinfection) was not yet confirmed.

Here, we test the hypothesis that P-Ps are more likely to encode ARGs than are other phages because they share characteristics of plasmids, such as presence of transposable elements and regions of high genetic plasticity. For this, we searched a large number of P-Ps, plasmids, and phages from reference databases for bona fide ARGs. We found many ARGs, and their acquisition seems to have been driven by transposable elements and integrons. To test whether the P-Ps can be induced, we scanned a collection of carbapenem-resistant strains for putative P-Ps. The tested cases showed an almost systematic induction of P-Ps. We then tested whether the induced P-Ps were able to convert a panel of sensitive strains into bacteria that were resistant to broad-spectrum cephalosporins.

## RESULTS

### Antibiotic resistance genes are common in phage-plasmids but are rare in other phages.

To quantitatively assess the distribution of ARGs in plasmids, phages, and P-Ps, we searched for these genes in the complete bacterial and phage genomes of the RefSeq database. For this, we first updated our database of P-Ps by using a previously described detection method (23) (Fig. S1A). The novel P-Ps were classified into groups based on their similarities to previously classified elements, as measured by the weighted gene repertoire relatedness (wGRR) (see Materials and Methods) (Fig. S1B). This led to an almost doubling of the database of P-Ps to a total of 1,416 P-Ps. These elements represent 5.6% of the 25,275 phages and plasmids.

10.1128/mbio.01851-22.6 FIG S1 Identification of phage-plasmids with antibiotic resistance genes. (A) 25,275 sequences of phages and plasmids that were retrieved from RefSeq in March of 2021, were searched for functions of P-Ps (see Materials and Methods). First, phage functions were annotated in plasmids. Second, the annotation profiles were evaluated by random forest classification models. In phages, we initially searched for plasmid features (partitioning, replication) and subsequently compared positive cases to known P-Ps and plasmids. All phages with plasmid genes having a higher similarity than wGRR = 0.4, were added to the P-P list. Overall, 1,416 P-Ps were detected including the P-Ps found in the first approach (see Materials and Methods). (B) All new detected P-P sequences were searched for homology to genomes of known P-Ps and were subsequently assigned to corresponding P-P groups (AB, SSU5 supergroup and groups, P1 group, N15 group). Assignment was done when a P-P had a wGRR value of ≥0.5, based on at least 50% of the genes, to a grouped P-P. (C) ARG-encoding plasmids, phage-plasmids, and phages detected by AMRFinderPlus. Download FIG S1, PDF file, 0.1 MB. Copyright © 2022 Pfeifer et al. 2022 Pfeifer et al. https://creativecommons.org/licenses/by/4.0/ This content is distributed under the terms of the Creative Commons Attribution 4.0 International license.

**FIG 1:**
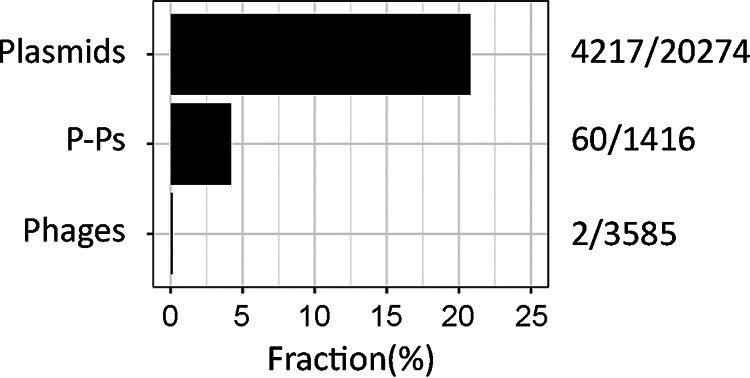
Number of mobile genetic elements encoding ARGs. The values after the bars indicate the number of elements encoding ARGs over the total number of elements considered in the analysis.

We searched for genes in phages, plasmids, and P-Ps with high sequence similarity (at least 99% identity and 99% coverage) to verified ARGs from three reference databases (ARG-ANNOT, ResFinder, and CARD). In agreement with previous studies (8), ARGs were frequently found in plasmids (20.8%) and were almost never found in phages (2 out of 3,585 genomes, <1‰) (Fig. 1). A total of 4.2% of the P-Ps carried ARGs, a frequency that is intermediate between that of phages (approximately 76.0 times greater) and plasmids (4.9 times lesser). To further validate the annotations of the ARGs, we compared the results of the three databases with the analysis of our data using the NCBI AMRFinderPlus software package (29). We found similar ARGs in P-Ps and phages as well as an increase in the number of plasmids with ARGs of about 13.5% (Fig. S1C). In P-Ps, the ARGs encode a variety of enzymes, such as β-lactamases, dihydrofolate reductases, and aminoglycosides-modifying enzymes. We also identified a few genes encoding efflux pumps (Table S1). Overall, our analysis shows that P-Ps encode ARGs much more often than do the remaining phages. In some cases, they encode resistance genes to last-line antibiotics, such as the *mcr-1* against colistin and various *blaKPC* (types 2, 3, 4, and 33) and *blaNDM-1* genes against carbapenems (Table S1).

10.1128/mbio.01851-22.1 TABLE S1 Antibiotic resistance genes detected in phage-plasmids. Download Table S1, XLSX file, 0.02 MB. Copyright © 2022 Pfeifer et al. 2022 Pfeifer et al. https://creativecommons.org/licenses/by/4.0/ This content is distributed under the terms of the Creative Commons Attribution 4.0 International license.

### Resistance genes are found in specific types and loci of phage-plasmids.

Most of the P-Ps carrying ARGs (47 of the 60 P-Ps) were found in the genomes of just four species: Acinetobacter baumannii (*n* = 8), Escherichia coli (*n* = 20), Klebsiella pneumoniae (*n* = 14), and Salmonella (spp. and *enterica*) (*n* = 5). This is not overly surprising; our previous study showed that these species had many P-Ps (23), many of the genomes of these species are available in the database, and these are all pathogenic bacteria that are known to develop antibiotic resistance (30). The majority of these P-Ps were assigned to well-defined P-P groups (23). P1-like P-Ps represent a third of the elements with ARGs (21 cases), of which all are in the P1 subgroup 1. We also detected 12 SSU5-related P-Ps and 8 AB-like P-Ps with ARGs (Table S1). Interestingly, we could not detect in P-Ps any ARGs belonging to the N15 group, the pMT1 group, or the P1 subgroup 2. The results for N15 are particularly intriguing because these elements are abundant in nosocomial species, such as E. coli and K. pneumoniae (23).

**FIG 2:**
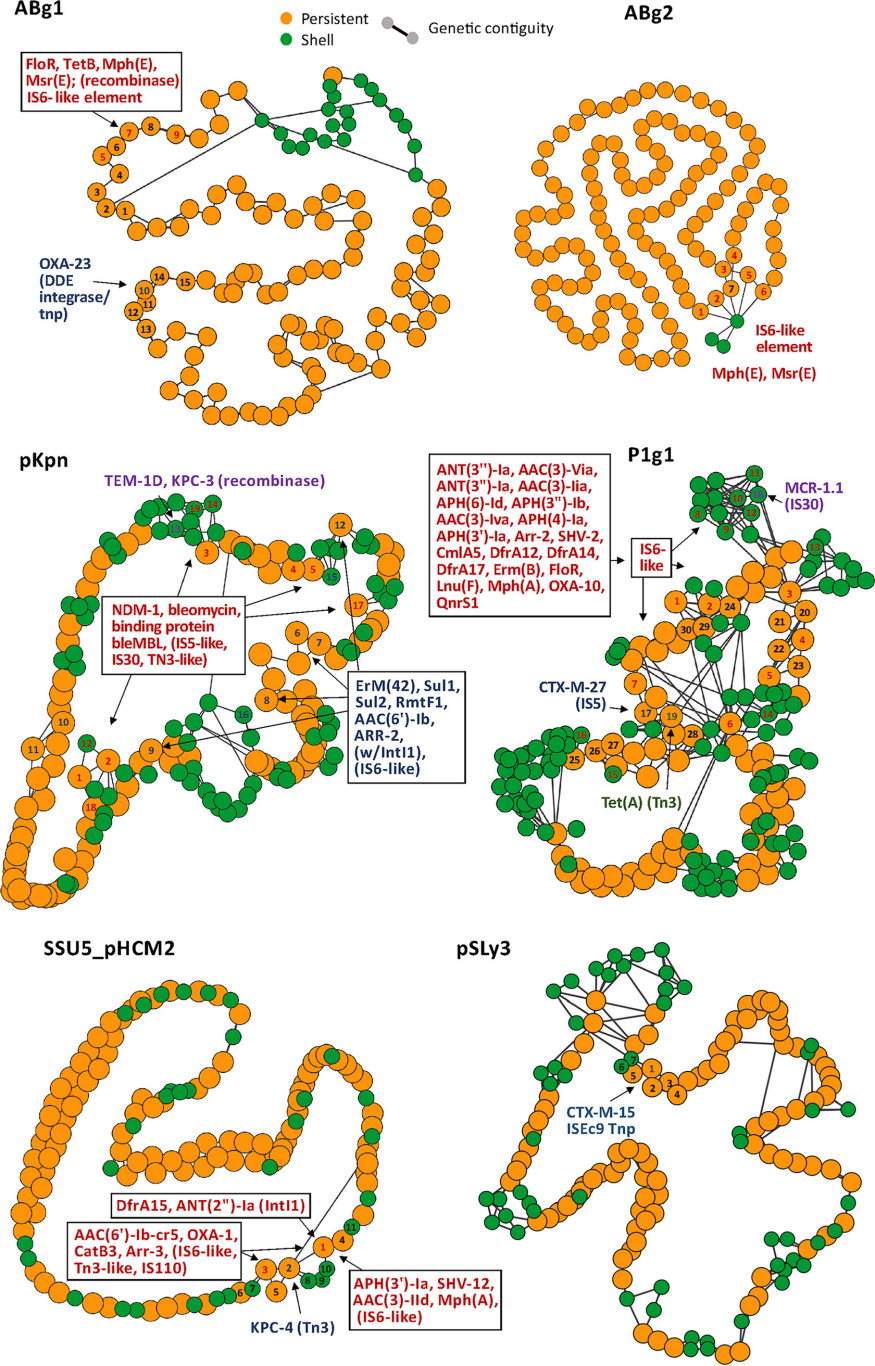
Genetic environments of ARGs in P-Ps’ pangenome graphs. Nodes are gene families. Genes (including ARGs) were grouped into a gene family (default parameters of PPanGGOLiN [31]) if they had a similarity of >80% identity covering at least 80% of the sequence. Orange: persistent, green: shell. Edges are shown for adjacent genes within the gene families (genetic contiguity). Gene families with colored numbers (red, blue, violet, green) are direct neighbors of ARGs containing genetic elements (transposon, IS, integron, recombinase [separated by colors]). Black numbers are given for proximal gene families. ABg1: 1-3,6,8-9:hypothetical, 4:nucleoside, 5:ATPase AAA, 7:3′–5′ exonuclease pyrophospho-hydrolase. ABg2: 1,3:hypothetical, 2,7:ribonucleoside-diphosphate red. 4:toprim domain protein, 5:ATPase AAA, 7:3′–5′ exonuclease pyrophosphohydrolase. SSU5_pHCM2: 1:PhoH, 2-5, 7-11:hypothetical, 6:DNA ligase. P1: 1:SSB, 2-5,15,17,19-20,23-24,28: hypothetical, 6:cell division inhibitor (Icd-like), 7-11:tail fiber, 12:recombinase, 13-14:tail fiber assembly, 16:ResMod subM, 18:Ref family, 21:bleomycin hydrolase, 22:transglycosylase, 25:DNA repair, 26:Phd/YefM (T-A), 27:doc (T-A), 29:lysozyme, 30:head processing. pKpn: 1:transcriptional regulator, 2,7,9,12-15,17-19:hypothetical, 3:phoH, 4:porphyrin biosynthesis protein, 5-6: AAA family ATPase, 8: ribonucl.-diphosphate reductase subunit 10–11:tail fiber domain-containing protein, 16:HsdR. pSLy3: 1:DUF3927 family, 2:tellurite/colicin resistance, 3-7:hypothetical.

We analyzed the genomic locations of ARGs in P-Ps to shed light on how these genes were acquired and how these events may have affected the genetic organization of P-Ps. For this, we computed the pangenomes of the P-P groups, selected gene families present in high or intermediate frequencies in the pangenomes to build a graph of the genetic organization of the elements, and placed the ARGs in relation to this backbone (Fig. 2). The pangenomes were divided into genes present in most strains (persistent), those present in few strains (cloud), or those of intermediate frequency (shell), according to (31). ARGs were never found in the persistent genomes of the P-Ps, concordant with the hypothesis that they were recently horizontally acquired and that they are not essential. Some P-Ps harbor one ARG, but the majority (*n* = 39) have multiple genes, with up to 13 ARGs being detected in a single putative P-P (pASP-135, NZ_CP016381) from the Aeromonas hydrophila strain AHNIH1 (Table S1). This fits previous suggestions that *Aeromonas* spp play a key role in the genetic transfer of ARGs (32). One of the ARGs is the *bla*_KPC-2_, which confers resistance to carbapenems and is of great concern, as it could act as a reservoir for this gene. Genes that commonly promote recombination and genomic plasticity, such as transposases and recombinases, were systematically identified in close proximity to the ARGs (Fig. S2). Transposases of the IS*6*-like family were particularly frequent next to the ARGs, especially those of the type IS*26* (Fig. 2). This family of ISs has been previously involved in the spread of clinically relevant ARGs, it commonly causes plasmid cointegration, and its insertion results in hybrid promoters that can influence the expression of neighboring genes (33). Notably, most ARG families (21 out of 24) of the P1 subgroup 1 are close to an IS*6*-like transposase (Fig. 2), suggesting that this transposable element drives the ARG acquisition in these P-Ps. In addition, in the AB, pKpn, and SSU5_pHCM2 groups, some ARGs are next to IS*5*-like, IS*30*-like, or Tn*3*-like transposases as well as several other types of recombinases. In the pSLy3-like group, we found no IS6-like transposases next to ARGs, but we did find an IS*Ec9* transposase (Fig. 2; Fig. S2).

10.1128/mbio.01851-22.7 FIG S2 Representative P-Ps with and without ARGs of each group. Genome-to-genome comparisons of P-Ps with and without ARGs. wGRR relatedness is indicated between the genomes. Assigned regions (red: directed, blue: inverse orientation) are showing the best-bidirectional-hits (BBHs), which are based on an all-versus-all protein sequence comparison (by MMseqs2 with at least 35% identity, 50% sequence coverage, and an E value of ≤1e^−4^ [see Materials and Methods]). Plasmid genes (partition and replication functions) are shown in orange, and phage functions (structure, lysis, packaging) are shown in blue, ARGs are shown in red, and genes assigned to transposases or IS elements are shown by green arrows. The names of the ARGs are shown in red in the order in which they appear. Download FIG S2, PDF file, 2.5 MB. Copyright © 2022 Pfeifer et al. 2022 Pfeifer et al. https://creativecommons.org/licenses/by/4.0/ This content is distributed under the terms of the Creative Commons Attribution 4.0 International license.

Interestingly, we found that ARGs tend to be present in a small number of loci in the genomes of P-Ps. Within the P1-like and the pKpn-like genomes, IS*6*-like transposable elements are inserted into a few distinct positions, whereas in the genomes of SSU5-like and AB-like P-Ps, all of the insertions are concentrated in just one locus (Fig. 2). This is in line with our previous finding that P1 and pKpn genomes are more plastic than those of the average P-Ps (they are more complex, have larger shell and cloud genomes, and more of plastic regions [23]). The conserved genes flanking the regions with ARGs often encode regulators, enzymes associated with DNA repair, or unknown functions. Few of them flank key, highly conserved phage functions.

Overall, this analysis revealed diverse classes of ARGs in different groups of P-Ps (Fig. 2; Fig. S2) that seem to have been acquired by the action of transposable elements.

### Integrons carrying ARGs are frequent in phage-plasmids.

**FIG 3:**
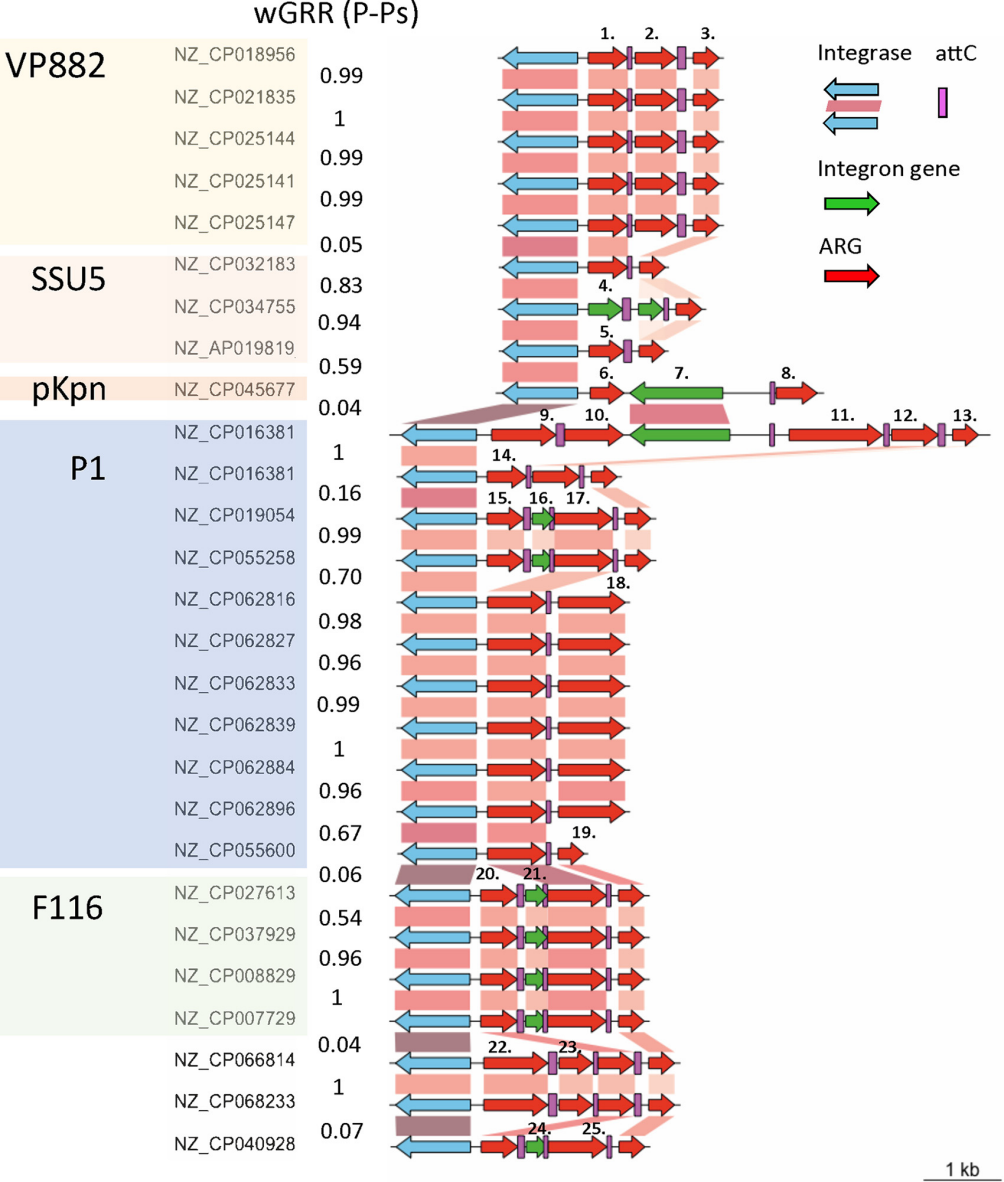
Integrons encoded in P-Ps. Genomic organization of integrons found in P-Ps, arranged by P-P groups and coaligned by the class I Intl1 integrase. The P-P type is highlighted by different colors. Gene-to-gene assignment is based on a BLASTP comparison when the alignment is at least 90% identical and covers at least 90% of the sequences. Blue gene arrows indicate the integrase genes, red arrows show the AMR genes (99% identity with 99% coverage to reference sequences), and green arrows represent the rest of the genes within the integrons. Numbers indicated above nonhomologous cassettes represent different types of ARGs: (1,14): *ant(2′')-Ia*; (2): *aac(6′)-33*; (3;13;19): *qacEΔ1*; (4): *aac(3)-Ib*; (5): *dfrA15*; (6): *arr-2*; (7): group II intron reverse transcriptase/maturase; (8): *aac(6′)-Ib*; (9): *bla*_CARB-2_; (10;17;25): *aadA2*; (11): *cmlA6*; (12): *catB11*; (15;20): *dfrA12*; (16;21;24): DUF1010 protein; (18): *aac(3)-VIa*; (22): *bla*_GES-1_; (23): *arr-6*.

Class 1 integrons are not mobile by themselves, but plasmids often carry such integrons with ARGs (resistance integrons) (34). A recent analysis identified more than 1,400 complete integrons in plasmids on the genome data set used in our study (35). In contrast, no integron carried by a phage was reported thus far. Accordingly, we searched for integrons in 3,585 phages lacking evidence of being P-Ps and found no single integron in these elements. Since P-Ps have characteristics that are intermediate between plasmids and phages, we screened them for integrons. We found 27 integrons in P-Ps. Integrons were especially abundant in P1-like elements (*n* = 11) (Fig. 3). Although the SSU5 supergroup has the most members (*n* = 268), just four P-Ps in this set were predicted to have integrons. Two dissimilar integrons were detected in just one P-P (NZ_CP016381) that was isolated from an A. hydrophila strain. Furthermore, the A. hydrophila P-P has some similarity to P1 (wGRR = 0.07, 19 homologous genes), albeit not enough to classify it as P1-like. 9 P-Ps with integrons were found in VP882-like and F116-like P-Ps.

These integrons have between two and five cassettes. Remarkably, nearly all of the genes within the cassettes were predicted to be ARGs (Fig. 3). As usual, *qacEdelta1*, which confers resistance to antiseptics, was detected in most integrons (20 out of 27) as a part of the 3′ conserved segment. We found 15 cooccurrences of this cassette with that of *aadA2* (aminoglycoside nucleotidyltransferase) and 10 with those containing *dfrA12*/*dfrA15* genes (trimethoprim resistance). A large diversity of other resistance genes was identified in integrons, including aminoglycosides modifying enzymes (*ant(2’’)-Ia*, *aac(6’)-33*, *aac(3)-Via*), rifampicin resistance genes (*arr-2*, *arr-6*), chloramphenicol resistance genes (*cmlA6*, *catB11*), and different β-lactamases, including the minor ESBL *bla*_GES-1_ and the penicillinase *bla*_CARB-2_ (Fig. 3). Hence, integrons in P-Ps encode a diverse panel of ARGs.

We compared the gene repertoire relatedness (wGRR) between integron-encoding P-Ps and the similarities between the integrons themselves. The gene cassette arrays tend to be highly similar when they are in the same type of P-P and highly distinct between unrelated P-Ps. However, in a few cases (pointed out by black arrows in Fig. 3), dissimilar P-Ps have similar integron cassettes (>90% identity and 90% coverage), suggesting an epidemic spread of genes providing a selective advantage (resistance to aminoglycosides and antiseptics). In all cases, the integrons of P-Ps had similar IntI1 type integrases. These results suggest that, as is the case for plasmids, type I integrons act as reservoirs for multiple ARGs in P-Ps.

### Phage-plasmids with resistance genes are induced by mitomycin C.

**FIG 4:**
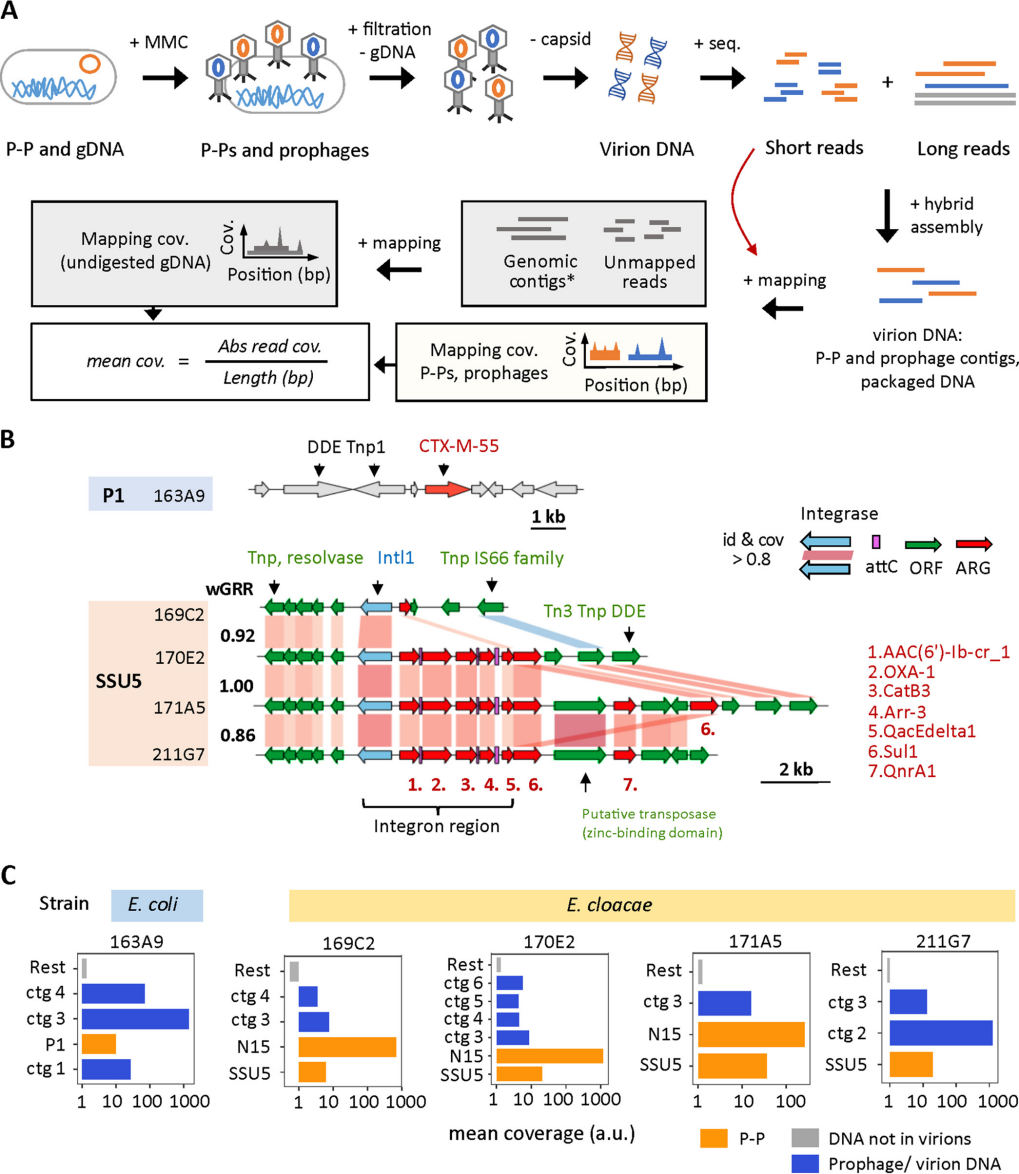
Induction of P-Ps and prophages in CRE strains. (A) CRE strains with ARG-encoding P-Ps were induced by 5 μg/mL MMC. 4 h after induction, phage particles were purified, and chromosomal DNA (gDNA) was removed by DNase I digestion. The phage capsid was degraded by proteinase K, and the virion DNA was purified and sequenced. The obtained short reads were coassembled with long reads from the genomic sequencing experiment (see Materials and Methods). The assemblies were compared to P-P and phage genomes and subsequently assigned. The read mapping coverage was computed (by mapping the short reads from the MMC experiment on them). The reads that did not map to the assemblies were used to compute the background coverage caused by the undigested gDNA (by mapping to genomic contigs obtained via the long read assembly). (B) In the genome of the P1-like P-P of the E. coli 163A9, the CTX-M-55 gene is found next to two DDE transposases. The ARGs encoded in the SSU5-like P-Ps from the E. cloacae strains 169C2, 170E2, 171A5, and 211G7 are in a complex region containing transposases and integrons. Homology assignments between P-Ps were done when the sequence similarity was at least 80% identity and covered 80% of the sequence of the gene (retrieved from an all-versus-all BLASTP comparison). The similarity between P-Ps is indicated by the weighted gene repertoire relatedness (wGRR). (C) The average read coverage was obtained and calculated as described in panel A. All contigs (= ctg) larger than 10 kb are shown. The coverage (a.u.: arbitrary unit) is plotted on a logarithmic *x* axis for the P-P contigs (P1 = P1-like, SSU5 = SSU5-like, N15 = N15-like) (orange), the contigs assigned to prophages or virion loaded DNA (blue), and the average background coverage (the rest of the coverage) obtained after mapping the remaining reads to genomic contigs (gray) for each tested CRE strain.

The recent acquisition of ARGs in P-Ps might make them inactive phages, as previously observed in a plasmid resembling a P1-like element (25), either because the insertion inactivates relevant functions or because natural selection of the bacterium could select for inactive elements. To test whether some ARG-encoding P-Ps are functional phages, we screened a collection of draft genomes of CRE strains for ARG-encoding P-Ps. We identified six strains (two E. coli and four E. cloacae) that we resequenced using long reads to obtain assemblies with higher quality (Fig. S3A). These genomes had six P-Ps with ARGs: one P1-like (of 163A9) in E. coli and five SSU5-like P-Ps, of which one was from E. coli (166F4) and four were from E. cloacae (169C2, 170E2, 171A5, 211G7) (Table S2). We then tested whether these P-Ps are induced by DNA damage by exposing the bacterial cells to mitomycin C (MMC). 3 to 4 h after MMC addition, a drop of cell-density indicated phage-dependent cell lysis (caused by the SOS response and consecutive prophage and/or P-P induction) (Fig. S3B). The phage particles were purified, and their DNA was extracted and sequenced by short reads (after the digestion of chromosomal DNA [gDNA]; see Materials and Methods). We then conducted hybrid assemblies by combining the short reads from the MMC experiment with the long reads from the genome sequencing (Fig. 4A).

10.1128/mbio.01851-22.2 TABLE S2 Phage-plasmids with ARGs in CRE strains. Download Table S2, XLSX file, 0.01 MB. Copyright © 2022 Pfeifer et al. 2022 Pfeifer et al. https://creativecommons.org/licenses/by/4.0/ This content is distributed under the terms of the Creative Commons Attribution 4.0 International license.

10.1128/mbio.01851-22.8 FIG S3 Genome sequencing and MMC induction experiments of CRE strains with ARG-encoding P-Ps. (A) The genomic DNA of the CRE strains with ARG-encoding P-Ps (163A9, 1664F4, 169C2, 170E2, 171A5, and 211G7) was isolated and sequenced using the PacBio long read technology. The obtained reads were assembled with flye, using the default parameters (see Materials and Methods). The scatterplots (upper panel) show the lengths of the contigs per strain, and the overall number is represented in bar plots (lower panel). (B) All CRE strains with P-Ps that have AMR genes were tested for inducibility by exposing them to 1 μg/mL and 5 μg/mL MMC in comparison to the control (no MMC). The growth experiments were conducted in 96-well plates, and each strain was tested twice (technical replicate). After the addition of MMC, growth was followed by the measurement of optical densities at 600 nm by a plate reader under shaking conditions at 37°C. (C) Short reads of the MMC induction experiment and long reads of the genome sequencing were used to conduct a coassembly with Unicycler (see Materials and Methods). Contig lengths are shown in the upper panel in the dot plots, with circular contigs in red and linear or unassigned contigs in black. The total contig number per strain is represented in bar plots in the lower panel. (D) Plasmid functions (partitioning, replication) are shown in orange, and phage genes (structure, packaging, lysis) are shown by blue arrows. The assignments are the BBHs that are based on an all-versus-all protein sequence comparison (by MMseqs2) and have at least 35% sequence similarity, 50% coverage (both sides), and an E value of ≤0.001. For the assignments, BBHs in the same orientation are shown in red, and inverses are shown in blue. Download FIG S3, PDF file, 2.7 MB. Copyright © 2022 Pfeifer et al. 2022 Pfeifer et al. https://creativecommons.org/licenses/by/4.0/ This content is distributed under the terms of the Creative Commons Attribution 4.0 International license.

For all of the P-Ps except for those of strain 166F4, the coassembly resulted in closed circular contigs or in larger assemblies with good homology to known P-Ps (Fig. S3C and D), which confirmed the structure of the replicons. This opened the possibility of studying the genetic context of the ARGs in these P-Ps. The P1-like P-P of the E. coli strain 163A9 contains two DDE transposases next to the β-lactamase encoded by a *bla*_CTX-M-55_ gene (Fig. 4B). The four SSU5-like P-Ps from E. cloacae are similar (wGRRs ranging from 0.86 to 1) (Fig. 4B; Fig. S3D), and their ARGs are in the same loci and in a complex region that includes various transposases and a type 1 integron with a highly similar integrase (identity and coverage >80%) (Fig. 4B). Three P-Ps contain integron regions with five ARGs, whereas the one from the strain 169C2 has a similar integrase gene but lacks gene cassettes (Fig. 4B). Downstream of the integron region a few (one to three) more ARGs are in a locus with multiple transposases. The number of ARGs of the SSU5-like P-Ps ranges from one (169C2) to eight (171A5), and they are predicted to encode resistance against several antibiotics, including penicillins (*bla*_OXA-1_), fluoroquinolones (*qnrA1*), aminoglycosides (*aac(6’)-Ib*) and sulfonamides (*sul1*) (Fig. 4B; Table S2).

To verify that P-P DNA is found in the virions, we mapped the short reads from the MMC experiment to the coassemblies and retrieved the average mapping coverage (Fig. 4A). The reads that were not mapped on the P-Ps were mapped on the genome contigs (see Materials and Methods) to obtain the relative frequency of the nondigested chromosomal DNA (background signal) (Fig. 4A). We considered that highly covered P-Ps and prophages, relative to the background chromosome, were induced and packaged into virions. Five of the six tested strains were indeed induced and produced viral particles with the ARG-encoding P-Ps (Fig. 4C). The DNA of these elements was present at diverse frequencies, possibly as a result of different burst sizes (which might be further affected by the presence of other prophages [36]). For one of the six strains (E. coli 166F4), we did not obtain any reads mapping to the P-P contig, suggesting that this SSU5-like P-P is inactive or is not inducible by MMC under our experimental conditions. Interestingly, in the genomes of three strains (E. cloacae 169C2, 170E2, and 171A5) we found two different types of P-Ps: the SSU5-like encoding the ARG and another P-P lacking ARGs that is related to the N15 group. Notably, we could also assign some of the sequences retrieved from the viral particles to chromosomal loci with prophages in the CRE strains, which shows that they were also induced by MMC (Fig. 4C; Table S3). The coverage of P-Ps and integrative prophages in the analysis of the viral particles is typically at least 1 order of magnitude higher than the average coverage of the background, showing that the result is not due to random contamination by bacterial DNA. Hence, most of the analyzed P-Ps, with or without ARGs, are inducible and are packaged into virions.

10.1128/mbio.01851-22.3 TABLE S3 VirSorter2 and wGRR analyses of coassembled contigs retrieved after MMC-induction experiments (including average read coverage). Download Table S3, XLSX file, 0.01 MB. Copyright © 2022 Pfeifer et al. 2022 Pfeifer et al. https://creativecommons.org/licenses/by/4.0/ This content is distributed under the terms of the Creative Commons Attribution 4.0 International license.

The MMC induction experiments confirmed that P-Ps with ARGs are inducible and are packaged into virions. Hence, these P-Ps confer antibiotic resistance to the bacterium and function as real phages. We then tested whether they are also capable of infecting, lysogenizing, and converting other host strains into becoming antibiotic resistant. Phages tend to have narrower host ranges than do conjugative plasmids, possibly because of their requirement for specific receptors at the cell envelope (37), the existence of numerous bacterial defenses against phages (38), and the presence of other prophages (39). Previous analyses suggest that temperate phages are able to infect only a small fraction of strains in a species (40, 41). Hence, the first challenge was to identify permissive hosts that were different from the strain carrying the P-P.

The four SSU5-like PPs are similar and presumably have similar host ranges. Since the requirements of host ranges of these phages are poorly understood, we tested 18 different E. cloacae strains retrieved from the Pasteur and the German collections (DSMZ) (Table S4). However, no lysogenic conversion was observed by the SSU5-like P-P.

10.1128/mbio.01851-22.4 TABLE S4 Bacterial strains that were used for infection experiments with ARG-encoding P-Ps. Download Table S4, XLSX file, 0.01 MB. Copyright © 2022 Pfeifer et al. 2022 Pfeifer et al. https://creativecommons.org/licenses/by/4.0/ This content is distributed under the terms of the Creative Commons Attribution 4.0 International license.

**FIG 5:**
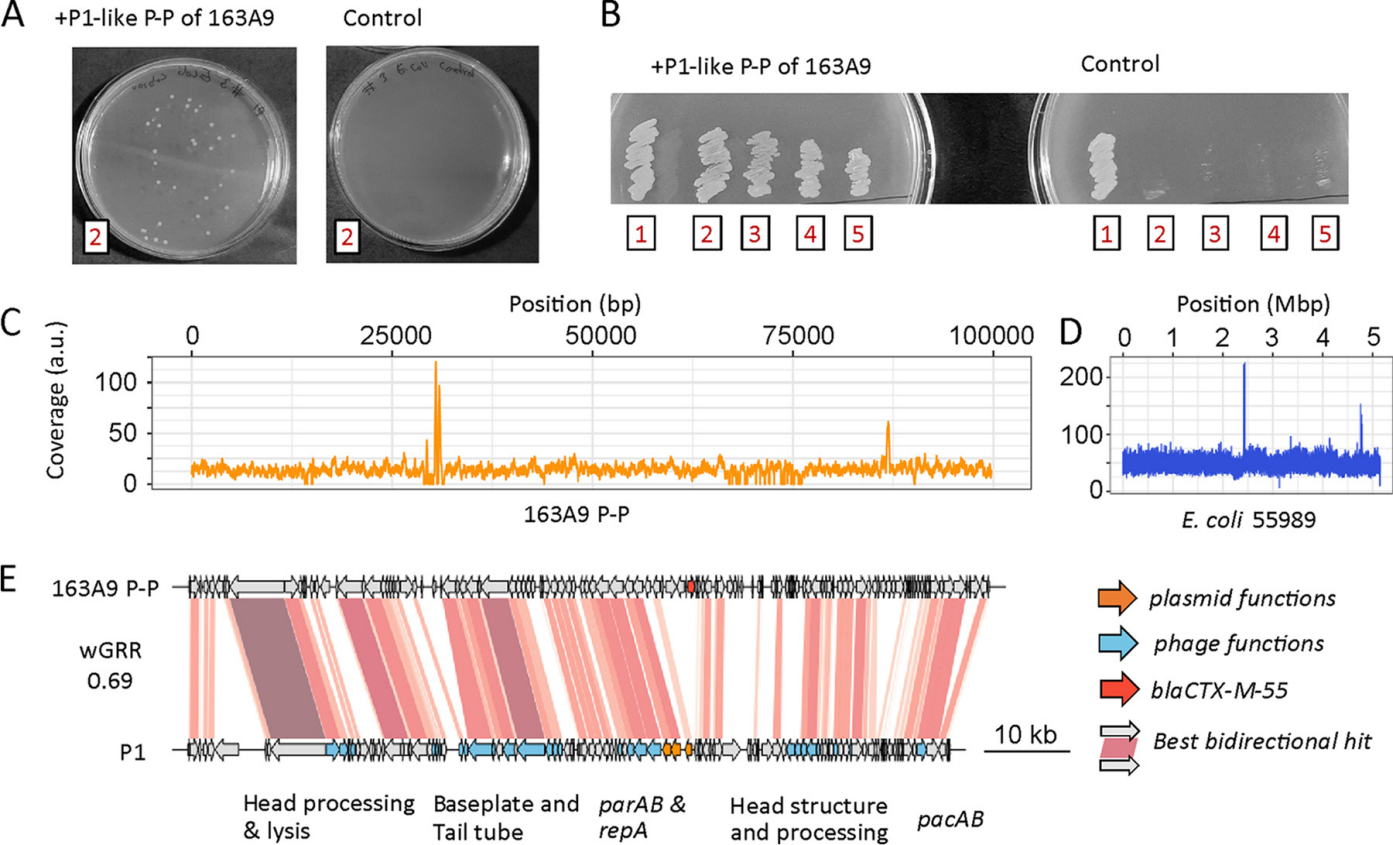
Lysogenic conversion of different E. coli strains (55989 [2], CIP 105917 [3], CIP 53.126 [4], and CIP 76.24 [5]) by the P1-like P-P of strain 163A9 [1]. (A and B) After the infection and plating experiment, four tested E. coli strains acquired resistance to carbenicillin. Examples of colonies of strain 55989 with the P1-like P-P of 163A9 are shown on LB agar plates with 100 μg/mL carbenicillin (left in panel A). Prior to infection, E. coli 55989 cells do not have the resistance (control, right in panel A). The original host of the P1-like P-P and all four lysogens are resistant to 100 μg/mL ampicillin (B). (C and D) Their genomes were isolated and sequenced by short reads (Fig. S12). The read coverage for the P-P genome (C) and the chromosomes of the host strain E. coli 55989 (D) are shown. (E) Genome comparison of the P-P from 163A9 and P1, coaligned to the first gene of P1. The alignment is matched with the read coverage plot in panel C. The functions of the P1 genes were retrieved from Łobocka et al. (78).

We chose a diverse panel of 12 E. coli strains and 1 S. enterica (CIP 82.29T) strain from the Pasteur collection to infect with the P1-like P-P (Table S4). The S. enterica strain was of particular interest to test the host range of the P-P. We then conducted infection experiments, in which we purified the phage particles, incubated them with the potential host strains, and screened them for resistant lysogens by plating the mixture on antibiotic-containing plates (here, carbenicillin; see Materials and Methods). For the S. enterica strain, we did not obtain lysogens. However, we found four different E. coli recipient strains (55989, CIP 105917, CIP 53.126, CIP 76.24), all from phylogroup B, that were initially sensitive but became resistant to carbenicillin after the infection with the P1-like P-P of strain 163A9 (Fig. 5A and B). The sequencing of the genomes of the recipient strains confirmed the acquisition of the ARG and of the rest of the P-P (Fig. 5C–E; Fig. S4A). Moreover, susceptibility tests with various β-lactam antibiotics (broad-spectrum penicillins, cephalosporins of different generations, carbapenems) confirmed the presence of the CTX-M-55 ESBL in the lysogenized strains. All four of the strains showed resistance to three broad spectrum penicillins (ticarcillin, piperacillin, amoxicillin) and a third-generation cephalosporin (cefotaxime) (Fig. S4B). Finally, we tested whether the P-P was fully functional as a phage by testing whether it could be induced in the new host and used to infect another cell. We exposed the E. coli 55989 strain with the P1-like P-P to MMC, purified the particles, and used them to infect the original antibiotic-sensitive E. coli 55989 strain. This revealed the acquisition of the P-P and the lysogenic conversion (Fig. S4C), thereby confirming that it is a fully functional phage. We can exclude the hypothesis that the resistance gene was transferred by other induced prophages (by generalized transduction) since (i) the complete P-P is detected in the new host strain with a homogenous read coverage (including the resistance gene), (ii) the viral particles of the two most highly induced integrative prophages should not be able to transfer the whole P-P because their genomes are much smaller (35 kb and 41 kb), (iii) the other prophages lack known ARGs, and (iv) in the new host, the P1-like P-P can be induced again and used to infect a sensitive host. This demonstrates that natural P1-like P-Ps can transfer ARGs as phages and can result in the lysogenic conversion of other strains.

10.1128/mbio.01851-22.9 FIG S4 Genome resequencing and antibiotic sensitivity tests of lysogens with the P1-like P-P of strain 163A9. (A) Genomes of all E. coli lysogens (CIP 105197, CIP 53.126, CIP 76.24, and 55989) having the P1-like P-P isolated from CRE strain 163A9 (including the original strain 163A9) were isolated and sequenced by short reads (Illumina). The read coverages for the P-P genome and the host chromosome are shown (55989: NC_011748, CIP 105917: NZ_CP041623, CIP 53.126: NZ_CP022959, CIP 76.24: NZ_CP009072). For the CRE strain 163A9, the mapping was done on the three largest assembled contigs (contig 1: 1,618,643 bp; contig 2: 1,508,600 bp; and contig 3: 1,662,471 bp). (B) Antibiotic susceptibility test of lysogens. E. coli lysogens (CIP 105197, CIP 53.126, CIP 76.24, 55989, CRE_163A9) having the P1-like P-P of strain 163A9 with the ARG *ctx-m-55* were tested for sensitivity against various β-lactam antibiotics (see Materials and Methods). (C) Reinduction and infection experimentation with the P1-like P-P from 163A9. (i) E. coli strain 163A9 was treated with MMC (see Materials and Methods) to induce the P1-like P-P. After 3 to 4 h, the phage particles were purified and were (ii) subsequently used to infect E. coli 55989. (iii) Lysogenized E. coli 55989 having the P1-like 163A9 P-P were exposed to MMC to induce the P-P again. The phage particles were purified, used to infect a nonlysogen variant of E. coli 55989, and plated on LB plates with 100 μg/mL carbenicillin to screen for lysogens. In the control, untreated E. coli 55989 cells were plated on LB plates with 100 μg/mL carbenicillin. Download FIG S4, PDF file, 2.0 MB. Copyright © 2022 Pfeifer et al. 2022 Pfeifer et al. https://creativecommons.org/licenses/by/4.0/ This content is distributed under the terms of the Creative Commons Attribution 4.0 International license.

## DISCUSSION

ARGs and integrons are commonly found in plasmids (8) but are rarely found in phages (16). P-Ps are temperate phages and plasmids. Here, we show that they are more much likely to encode ARGs than are the other phages. We also show that they frequently encode integrons with ARGs, which had never been observed in functional phages. We demonstrate that one of the P-Ps was a fully functional phage that could be induced and produced lysogens resistant to broad-spectrum cephalosporins. This could be shown for two full cycles of induction, infection and conversion. We are confident that the elements we are investigated are true P-Ps because this claim is supported by several independent lines of evidence. First, long read sequencing failed to show any P-Ps in the long (>1 Mb) chromosomal contigs. Second, the hybrid assembly recovered one single circularized contig for all of the P-Ps, except for one SSU5-like P-P of strain 170E2. Third, sequences homologous to known P-Ps were detected only on nonchromosomal contigs (with sizes between 20 kb and 100 kb). Finally, the P-Ps here investigated are all related to known P-Ps, such as P1, N15 or SSU5. So far, these types of P-Ps were found to be exclusively extrachromosomal in the many thousands of complete genomes we have investigated.

Nevertheless, there are some limitations to our study. Some P-Ps may not be inducible by MMC or may be inactive, a trait common among integrative prophages (42). We tested six strains that had nine putative P-Ps, and all but one of them could be induced to produce viral particles containing phage DNA. This suggests that many of the P-Ps are still functional. Still, we could not obtain new lysogens for a group of closely related SSU5-like P-Ps by screening for antibiotic resistance. This could be due to a lack of expression (or function) of the ARGs. We tried to minimize this problem by using strict criteria in the identification of the ARGs (99% identity over 99% sequence coverage to known ARGs), but their expression may depend on the genetic background. The lack of lysogens may also be caused by bacterial resistance to phage infection. Previous works have shown that the interaction matrices of bacteria with temperate phages tends to be sparse (40). That is, most combinations do not result in a productive infection. This is because many bacteria have anti-phage systems, lack appropriate cell receptors, or have phages repressing infecting P-Ps (39). In addition, the replication initiators of P-Ps may be incompatible with those of resident plasmids, preventing their establishment in lysogens. Further work will be needed to explore the host range of P-Ps carrying ARGs. In contrast, we found multiple E. coli strains susceptible to the P1-like phage of strain 163A9. These differences may be associated with the host range of the P-Ps, which is known to be unusually broad in P1 (43, 44).

A few previous reports identified ARGs in P-P-like elements among Enterobacteria, even though evidence of induction is often lacking (25, 26, 45). In our study, we show that this is a general trend not only of P1-like elements but also of other types of P-Ps. Such cases can be found in other bacterial clades of important nosocomial pathogens, such as Acinetobacter. Overall, they are much more likely to carry ARGs than the other phages. We show that they carry a wide diversity of resistances. Most worrisome, many clinically relevant ARGs are found in P-Ps, including the carbapenemase genes *bla*_KPC-3_ and *bla*_NDM-1_. The *bla*_KPC_-like genes are involved in the diffusion of carbapenem resistance in Italy, Israel, and the USA, whereas the *bla*_NDM_-like gene is disseminated worldwide (46). Among the last-resort antibiotics, colistin was reintroduced in the armamentarium to fight against carbapenem-resistant Gram-negative rods, despite its nephrotoxicity. Plasmid-mediated colistin resistance has been recently described (47). This ARG, named *mcr-1*, was also identified in P-Ps. Beyond these two important resistance mechanisms, the *rmtF* gene is of further importance and is also carried by P-Ps. This gene encodes a 16S RNA methyltransferase conferring resistance to almost all of the aminoglycosides used for treatment (48). Thus, P-Ps are involved in the diffusion of resistance to the main antibiotic families, including β-lactams (e.g., *bla*_KPC-3_), aminoglycosides, fluoroquinolones (e.g., *qnrA1*), and polymyxins.

The presence of ARGs in P-Ps raises concerns that are common to the resistances found in other plasmids, notably that they can be spread across bacterial populations. However, the fact that P-Ps are also phages raises additional concerns. First, unlike conjugative plasmids, phages transfer their DNA in viral particles and do not require direct contact between cells for the transfer. Hence, they can transfer between bacteria present in different times and places. Second, the lytic cycle of phages amplifies their genomes hundreds of times (e.g., 400 for P1 [49]) for packaging in the viral particles, which may result in bursts of transfers of ARGs. The process of phage replication in the cell could also lead to the overexpression of ARGs, which could liberate enzymes that detoxify the environment for the remaining bacteria, a process akin to the production of the Shiga toxin from the prophages encoding it (50). However, it must be stated that in our experiences the only drug that induced P-Ps was MMC. Finally, P-Ps are more likely to recombine with other phages than with the remaining plasmids because they share numerous homologous genes (23). This may pose a threat of the transfer of ARGs to other types of phages.

Plasmids are known to contain many transposable elements and integrons that facilitate the translocation of ARGs within and between replicons (9). In contrast, phages typically have few (if any) such elements (51). Here, we show that P-Ps have numerous transposable elements associated with ARGs and integrons. Hence, P-Ps can take advantage of genetic elements typical of plasmids, transposable elements, and integrons to acquire ARGs that can then be spread horizontally by viral particles. Integrons are reservoirs of ARGs and, especially in clinical settings, promote the spread of multidrug resistance (52). Their identification in P-Ps is worrisome because the ability of the integron to incorporate novel cassettes from other integrons implies that upon the acquisition of an integron, the repertoire of ARGs of the P-Ps can evolve faster to incorporate novel types of resistance.

This raises the question of why P-Ps have so many more ARGs than do other phages. About half of the sequenced phages are virulent (53). They are not expected to carry ARGs because they do not produce lysogens, although this cannot be completely excluded, since they may produce pseudolysogens (54). The causes of the differences in frequencies of ARGs in P-Ps and other temperate phages are less obvious. It could be argued that the frequency of ARGs in P-Ps is caused by their abundance in bacterial pathogens. However, most sequenced phages in the database are also from a few genera, though many of them are Enterobacteria, and this does not seem sufficient to explain the difference in the number of ARGs present in the two types of elements. For example, the naturally occurring integrative prophages of E. coli analyzed in this study are completely devoid of identifiable ARGs. We propose that the differences between the P-Ps and the integrative, temperate phages result from a combination of factors. First, P-Ps tend to be larger than other phages (23). This is particularly relevant for phages because they can only package an excess of a few percent of their genome size. A sudden large increase in genome size precludes the packaging of the genome and thus blocks phage transfer. Hence, a genome of 49 kb, like phage lambda, can only accommodate small insertions. In contrast, P1-like elements are on average 96 kb (23) and can tolerate larger changes. Since the integration of ARGs in P-Ps involves the transposition of the gene, flanking transposable elements, and/or integrons, these insertions may be too large for most integrative phages. Accordingly, we found ARGs in the largest P-Ps, such as the P1-like and the SSU5-like, but not in the much smaller N15-like elements (average of 55 kb, [23]). The differences between the P-Ps and the other phages in relation to the presence of ARGs could also be caused by the regions of homology to the plasmids of the former. They might facilitate genetic exchanges between plasmids and P-Ps.

Independently of the reasons leading to an overrepresentation of ARGs in P-Ps relative to the other phages, the subsequent evolution of the loci containing them in P-Ps may result in streamlined, compact loci that could be easier to transfer to other phages. Indeed, integrative temperate phages of pathogenic bacteria encode many virulence factors, and we cannot find a reason why, given enough time, they will not eventually acquire ARGs. This would be the most worrisome outcome of the recent evolutionary process of the acquisition of ARGs by human-associated bacteria, since, as stated above, phages are extremely abundant, spread rapidly in the environment, and can infect bacteria in different geographical locations and at different times.

## MATERIALS AND METHODS

### Genomic data.

We used the completely assembled genomes of 8,399 bacterial strains, including their 21.550 plasmids, and the completely assembled genomes of 3,725 phages. All genome data were retrieved from the nonredundant NCBI RefSeq database (55) (March 2021).

### Similarity between mobile genetic elements.

The weighted gene repertoire relatedness (wGRR) assesses the similarity of gene repertoires between pairs of mobile genetic elements by taking into account their numbers of bi-directional best hits (BBH) and their sequence identities. It is computed as described previously (23) for all genomes and contigs of phages, plasmids, and P-Ps. Briefly, MMseqs2 (v. 13-45111) (56) was used to conduct all-versus-all gene comparisons between the elements. The BBHs between two genomes were extracted if they met the following criteria: E value of <10^−4^ and a sequence identity of >35%, covering at least 50% of both gene sequences. The computation of the wGRR follows.

$$\mathrm{wGRR}\;\left(A,B\right)=\frac{\sum_{i}^{P}id(A_{i},B_{i})}{\min(\#A,\#B)}$$

A_i_ and B_i_ are the *i*th BBH pair of the *P* total pairs. The gene number of the smaller genome is min(#A,#B), and the sequence identity between the BBH pair is id(A_i_,B_i_). The sum of the sequence identities (of the BBHs) normalized to the gene number of the smaller genome is defined as the wGRR between the two genomes.

### Identification and classification of phage-plasmids (P-Ps).

P-P genomes were identified as described previously (23). Briefly, we searched for genes encoding phage-like functions in plasmids of intermediate size (>10 kb and <300 kb) by using carefully selected pVOG (57), PFAM (58), and TIGRFAM (59) HMM protein profiles with HMMER v 3.3.2 (60). We only analyzed plasmids larger than 10 kb because no dsDNA phage in RefSeq NCBI is smaller than 10 kb. This is less than the usual size limit for prophage identification (61). The maximal threshold of 300 kb was set to minimize false-positives that could have arisen by considering megaplasmids that are actually secondary chromosomes and may thus be targeted by temperate phages for integration (62). These thresholds led to the rejection of 23.0% (<10 kb) and 6.6% (>300 kb) of the elements annotated as plasmids. A positive hit was assigned if the alignment covered at least 50% of the protein profile with a domain i-E value of <10^−3^. The distributions of hits in the plasmids were given to previously trained random forest models that provided the list of putative P-Ps. All dsDNA phages (larger than 10 kb) were screened for plasmid functions using protein profiles specific for plasmid replication and partition systems (63). No phage larger than 300 kb was predicted to be a P-P. Phages with hits for plasmid functions were extracted and were compared with the plasmids and the P-Ps (23). Novel elements having a wGRR of ≥0.4 with elements present in the list of previously identified P-Ps were added to the list of putative P-Ps. This resulted in 1,416 putative P-Ps, 740 of which were previously identified.

The classification of novel P-Ps is based on the similarity to previously identified P-P groups. P-Ps that were not identified in our previous study (23), typically because they correspond to more recent genome sequences, were assigned to defined P-P groups when they had wGRR values of ≥0.5 and at least half of their genes were homologous to a previously classified P-P. When there were multiple hits, the P-P was classified according to the classification of the element with the highest wGRR.

### Identification of antibiotic resistance genes (ARGs), IS elements, and integrons.

We searched genomes for ARGs using the CARD (64), ResFinder (65), and ARG-ANNOT (66) databases as references. We searched for sequence similarity between the genes of a MGE (phage, plasmid, P-P) and these databases using BLASTP (v.2.12.0+) (67) (to compare with the protein sequences of CARD and ARG-ANNOT) and BLASTX (v.2.12.0+) (67) (for the nucleotide sequences of ResFinder). We collected all hits in all databases, respecting the following constraints: E value of <10^−5^, sequence identity of ≥99%, and alignment covering sequences by ≥99%. The results were compared with the output of AMRFinderPlus (3.10.18) (tool from NCBI for ARG detection [29]) (Fig. S1C). IS elements were identified using ISEScan (v. 1.7.2.3, default parameters) (68). Integrons were identified using IntegronFinder (v. 2.0rc6, default parameters) (35).

### Pangenome graphs.

To compute the pangenomes of the P-P groups (including newly assigned members), we followed the same workflow as described previously (23). We computed the pangenome with PPanGGOLiN (v. 1.1.136, default parameters) (31). Genes (including ARGs) were grouped into a gene family if they had an identity of at least 80% covering 80% of the sequence. We made a visualization of the pangenome graphs with Gephi (https://gephi.org/) and igraph (https://igraph.org/r/) in the R environment.

### ARG-encoding P-Ps in carbapenem-resistant *Enterobacteriaceae*.

The draft genomes of 3,324 carbapenem-resistant Enterobacterales (CRE) received from the French National Reference center were screened for ARG-containing P-Ps. For this, we predicted the genes using Prodigal (v2.6.3, with default parameters) (69) and compared each contig with known P-Ps using the wGRR. We selected contigs with at least 25 genes and a wGRR of ≥0.4 for further study. These contigs were annotated in terms of ARGs, using the same method as that used for the P-Ps (see the subsection on the identification of ARGs).

Strains with contigs that were regarded as parts of putative P-Ps and encoding ARGs were then resequenced using long reads to ensure that the genome of the putative P-P was indeed as predicted and that it was extrachromosomal. Cells were cultivated in 4 mL LB-Miller medium (with 50 μg/mL carbenicillin at 37°C and 250 rpm) for ~16 h and pelleted. Their DNA were isolated with a modified version of the guanidium thiocyanate method (prior to DNA precipitation, the samples were treated with RNase A at 37°C for 30 min) (70). DNA library preparation (SMRTBell Library 10 kb insert size) and sequencing were done with the Biomics sequencing platform of the Institut Pasteur (C2RT) (Paris, France) with the technology of Pacific Biosciences. The obtained reads were assembled by flye (v.2.7.1-b1590) (71), using the default parameters (Fig. S3A).

### Growth experiments with mitomycin C (MMC).

The CRE strains with ARG-encoding P-Ps were first cultivated in 4 mL LB-Miller medium with 50 μg/mL carbenicillin at 37°C for ~16 h. The stationary cultures were diluted 1:100 and cultivated in a 96-well plate (200 μL per well) in LB-Miller medium with 50 μg/mL carbenicillin for 1 h. Subsequently, mitomycin C (MMC) (Sigma-Aldrich, St. Louis, United States) was added in final concentrations of 5 μg/mL, 1 μg/mL, and without MMC. Growth was monitored by following the absorbance at OD_600_ measured with a TECAN GENios plate reader (Männedorf, Switzerland) (Fig. S3B).

### Polyethylene glycol (PEG) precipitation of phage virions.

CRE strains with P-Ps were cultivated as described in the MMC growth experiment. 4 mL cultures were started in LB-Miller with 50 μg/mL carbenicillin by using 1:100 dilutions of the overnight cultured strains. 5 μg/mL MMC was added after 1 h. 4 h after the MMC addition, samples (2 mL) were taken for PEG precipitation and pelleted. The supernatant was filtered (0.22 μm) (phage lysate). To these phage lysates, a 5× PEG/NaCl (PEG-8000 20%, NaCl 2.5 M) solution was added in a 1:5 ratio, inverted several times, and chilled on ice for 1 h. Subsequently, the virions were pelleted at 3 min and 13,000 × *g*, and the supernatant was carefully discarded. The pellets were resolved in TBS buffer (50 mM Tris-HCl [pH 7.5], 150 mM NaCl) in 1/10 of the initial phage lysate volume and were incubated for another hour on ice. The PEG precipitated samples were further used for phage DNA extraction or infection experiments.

### Extraction and sequencing of DNA located in virions.

Virion DNA was extracted as described by Jakočiūnė and Moodley (72) after PEG precipitation, starting from step 3.2. Residual bacterial DNA was removed by treating samples with DNase I and RNase at 37°C. The phage protein capsid was digested with Proteinase K, and the DNeasy blood and tissue kit (Qiagen, Hilden, Germany) was used to purify the DNA. The quantity and quality of the purified DNA was checked by a Qubit fluorometer and a NanoDrop spectrometer. Library preparation (Illumina TruSeq DNA PCR-Free), sequencing, and quality checks were done on the Biomics sequencing platform of the Institut Pasteur (C2RT) (Paris, France) by short reads (paired-end, 250 bp length) on a MiSeq system (Illumina, San Diego, U.S.).

### Sequence data processing.

We took the DNA obtained after the MMC induction experiment and tried to assemble the P-Ps. However, given the presence of repeats in these elements, they were not fully assembled. To obtain the complete sequences, we put together the long reads from the genome sequencing (obtained before) and the short reads from the MMC induction experiment (see the previous subsection). These were then coassembled using Unicycler (v. 0.4.8) (73) with default parameters. The hybrid assembly resulted in 4 to 15 linear and/or circular contigs per strain representing the sequences of the induced P-Ps, prophages, and other DNA found in virions after MMC treatment (Fig. S3C). We evaluated the assemblies by checking whether the P-P contigs were closed (fully assembled) or not by comparing them with known P-Ps (Fig. S3D). Subsequently, we mapped the reads (obtained after the MMC induction) on these assemblies to assess how they cover them using bowtie2 (v. 2.4.4) (74) with the default parameters. To extract the coverage, we converted the output SAM files to a sorted BAM file using SAMtools (v. 1.13) (75) and obtained the coverage with BEDTools (v2.30.0) (76). In addition, we computed the (background) coverage caused by undigested gDNA. For this, we took the short reads (from the MMC induction experiment) that did not map to the hybrid assembled contigs (DNA outside virions) and aligned them with the contigs acquired from the genome sequencing experiment. The mean coverage was computed by dividing the absolute read coverage per contig (genome) by the size of the contig (genome).

### Generation of antibiotic resistant phage-plasmid lysogens.

PEG precipitated phage lysates were prepared and stored at 4°C. Potential host strains were cultivated the day before in 4 mL LB-Miller medium for approximately 16 h at 37°C and 250 rpm. The stationary cultures were diluted 1:100 in LB-Miller medium and grown until an OD_600_ of 0.5 to 1. Subsequently, 50 μL of the phage lysate was added to 50 μL of the host culture with 2 mM CaCl_2_ and was incubated under nonshaking conditions at 37°C for 1 h. After incubation, the cell/phage-lysate mixture was plated on agar plates at the required antibiotic concentration (to screen for lysogens). Antimicrobial susceptibility tests were performed as described (77) and interpreted according to the EUCAST guidelines. Colonies were tested by polymerase chain reaction (PCR) for the presence of P-P genomes (amplifying two regions: for region 1 with 522 bp 163A9-P1-R1A 5′-CTACCAGACCGCCTTTCTCAAAC-3′, 163A9-P1-R1B: 5′-TTGCCGAAACTAGAGAATAAATACGG-3′, and for region 2 with 423 bp 163A9-P1-R2A 5′-TTAACCTTTGTCGGCGTCGG-3′, 163A9-P1-R2B 5′-ATGTCATTCTTTTCTACATTAAAAACAGC-3′). Because the PCR can lead to false-positives, we confirmed the presence of the P-Ps by resequencing the whole-genome (P-P and host chromosome). The genomic DNA was isolated as described in the subsection on ARG-encoding P-Ps in CRE strains. Library preparation (Illumina TruSeq DNA PCR-Free) and sequencing were done on the C2RT Biomics platform of the Institut Pasteur (using short reads, paired-end, 250 bp) on an Illumina MiSeq system. The resulting reads were mapped on the P-Ps and on the host genomes (E. coli 55989: NC_011748, E. coli CIP 105917: NZ_CP041623, E. coli CIP 53.126: NZ_CP022959, E. coli CIP 76.24: NZ_CP009072). Coverage was extracted as described in the subsection on processing sequencing data.

### Data processing, storage and availability.

If not otherwise stated, all analysis and illustrations were done in the R environment (https://www.r-project.org/) with RStudio (v. 1.4.1106). All reads were uploaded to the European Nucleotide Archive (ENA) (https://www.ebi.ac.uk/ena). Short and long reads from the MMC induction, the genome sequencing experiment, the verification of P-P acquisition, and the sequences gained by the genome and hybrid assemblies are accessible under ENA study number PRJEB52357. Details on read accession numbers, assemblies, and experiments are listed in Table S5.

10.1128/mbio.01851-22.5 TABLE S5 Accession numbers of reads from the genome sequencing, MMC induction experiments (hybrid assembly included) and the resequencing of lysogens. Download Table S5, XLSX file, 0.01 MB. Copyright © 2022 Pfeifer et al. 2022 Pfeifer et al. https://creativecommons.org/licenses/by/4.0/ This content is distributed under the terms of the Creative Commons Attribution 4.0 International license.

## References

[1] Hernando-Amado S, Coque TM, Baquero F, Martínez JL. 2019. Defining and combating antibiotic resistance from One Health and Global Health perspectives. Nat Microbiol 4:1432–1442. doi:10.1038/s41564-019-0503-9. 31439928

[2] Spera AM, Esposito S, Pagliano P. 2019. Emerging antibiotic resistance: carbapenemase-producing enterobacteria. Bad new bugs, still no new drugs. Infez Med 27:357–364. 31846984

[3] Lin Q, Wang Y, Yu J, Li S, Zhang Y, Wang H, Lai X, Liu D, Mao L, Luo Y, Tang G, Chen Z, Sun Z. 2021. Bacterial characteristics of carbapenem-resistant *Enterobacteriaceae* (CRE) colonized strains and their correlation with subsequent infection. BMC Infect Dis 21:638. doi:10.1186/s12879-021-06315-0. 34215214 PMC8254368

[4] Worthington RJ, Melander C. 2013. Overcoming resistance to β-lactam antibiotics. J Org Chem 78:4207–4213. doi:10.1021/jo400236f. 23530949 PMC3644377

[5] Bonnin RA, Jousset AB, Emeraud C, Oueslati S, Dortet L, Naas T. 2020. Genetic diversity, biochemical properties, and detection methods of minor carbapenemases in Enterobacterales. Front Med (Lausanne) 7:616490. doi:10.3389/fmed.2020.616490. 33553210 PMC7855592

[6] Che Y, Yang Y, Xu X, Břinda K, Polz MF, Hanage WP, Zhang T. 2021. Conjugative plasmids interact with insertion sequences to shape the horizontal transfer of antimicrobial resistance genes. Pnas 118:e2008731118. doi:10.1073/pnas.2008731118. 33526659 PMC8017928

[7] Carattoli A. 2013. Plasmids and the spread of resistance. Int J Med Microbiol 303:298–304. doi:10.1016/j.ijmm.2013.02.001. 23499304

[8] Partridge SR, Kwong SM, Firth N, Jensen SO. 2018. Mobile genetic elements associated with antimicrobial resistance. Clin Microbiol Rev 31:e00088-17. doi:10.1128/CMR.00088-17. 30068738 PMC6148190

[9] Yao Y, Maddamsetti R, Weiss A, Ha Y, Wang T, Wang S, You L. 2022. Intra- and interpopulation transposition of mobile genetic elements driven by antibiotic selection. Nat Ecol Evol 6:555–564. doi:10.1038/s41559-022-01705-2. 35347261 PMC12520065

[10] Deng Y, Bao X, Ji L, Chen L, Liu J, Miao J, Chen D, Bian H, Li Y, Yu G. 2015. Resistance integrons: class 1, 2 and 3 integrons. Ann Clin Microbiol Antimicrob 14:45. doi:10.1186/s12941-015-0100-6. 26487554 PMC4618277

[11] Mazel D. 2006. Integrons: agents of bacterial evolution. Nat Rev Microbiol 4:608–620. doi:10.1038/nrmicro1462. 16845431

[12] Chiang YN, Penadés JR, Chen J. 2019. Genetic transduction by phages and chromosomal islands: the new and noncanonical. PLoS Pathog 15:e1007878. doi:10.1371/journal.ppat.1007878. 31393945 PMC6687093

[13] Brüssow H, Canchaya C, Hardt W-D. 2004. Phages and the evolution of bacterial pathogens: from genomic rearrangements to lysogenic conversion. Microbiol Mol Biol Rev 68:560–602, table of contents. doi:10.1128/MMBR.68.3.560-602.2004. 15353570 PMC515249

[14] Volkova VV, Lu Z, Besser T, Gröhn YT. 2014. Modeling the infection dynamics of bacteriophages in enteric *Escherichia coli*: estimating the contribution of transduction to antimicrobial gene spread. Appl Environ Microbiol 80:4350–4362. doi:10.1128/AEM.00446-14. 24814786 PMC4068684

[15] Borodovich T, Shkoporov AN, Ross RP, Hill C. 2022. Phage-mediated horizontal gene transfer and its implications for the human gut microbiome. Gastroenterol Rep (Oxf) 10:goac012. doi:10.1093/gastro/goac012. 35425613 PMC9006064

[16] Enault F, Briet A, Bouteille L, Roux S, Sullivan MB, Petit M-A. 2017. Phages rarely encode antibiotic resistance genes: a cautionary tale for virome analyses. ISME J 11:237–247. doi:10.1038/ismej.2016.90. 27326545 PMC5315482

[17] Calero-Cáceres W, Ye M, Balcázar JL. 2019. Bacteriophages as environmental reservoirs of antibiotic resistance. Trends Microbiol 27:570–577. doi:10.1016/j.tim.2019.02.008. 30905524

[18] Colavecchio A, Cadieux B, Lo A, Goodridge LD. 2017. Bacteriophages contribute to the spread of antibiotic resistance genes among foodborne pathogens of the *Enterobacteriaceae* family – a review. Front Microbiol 1108. 10.3389/fmicb.2017.01108 PMC5476706 28676794

[19] Brown-Jaque M, Calero-Cáceres W, Muniesa M. 2015. Transfer of antibiotic-resistance genes via phage-related mobile elements. Plasmid 79:1–7. doi:10.1016/j.plasmid.2015.01.001. 25597519

[20] Howard-Varona C, Hargreaves KR, Abedon ST, Sullivan MB. 2017. Lysogeny in nature: mechanisms, impact and ecology of temperate phages. ISME J 11:1511–1520. doi:10.1038/ismej.2017.16. 28291233 PMC5520141

[21] Kim M-S, Bae J-W. 2018. Lysogeny is prevalent and widely distributed in the murine gut microbiota. ISME J 12:1127–1141. doi:10.1038/s41396-018-0061-9. 29416123 PMC5864201

[22] Tuttle MJ, Buchan A. 2020. Lysogeny in the oceans: lessons from cultivated model systems and a reanalysis of its prevalence. Environ Microbiol 22:4919–4933. doi:10.1111/1462-2920.15233. 32935433

[23] Pfeifer E, Moura de Sousa JA, Touchon M, Rocha EPC. 2021. Bacteria have numerous distinctive groups of phage-plasmids with conserved phage and variable plasmid gene repertoires. 5. Nucleic Acids Res 49:2655–2673. doi:10.1093/nar/gkab064. 33590101 PMC7969092

[24] Sternberg N. 1990. Bacteriophage P1 cloning system for the isolation, amplification, and recovery of DNA fragments as large as 100 kilobase pairs. Proc Natl Acad Sci USA 87:103–107. doi:10.1073/pnas.87.1.103. 2404272 PMC53208

[25] Zhou W, Liu L, Feng Y, Zong Z. 2018. A P7 phage-like plasmid carrying *mcr-1* in an ST15 *Klebsiella pneumoniae* clinical isolate. Front Microbiol 9:11. doi:10.3389/fmicb.2018.00011. 29403463 PMC5786510

[26] Yang L, Li W, Jiang G-Z, Zhang W-H, Ding H-Z, Liu Y-H, Zeng Z-L, Jiang H-X. 2017. Characterization of a P1-like bacteriophage carrying CTX-M-27 in *Salmonella spp.* resistant to third generation cephalosporins isolated from pork in China. Sci Rep 7:40710. doi:10.1038/srep40710. 28098241 PMC5241659

[27] Billard-Pomares T, Fouteau S, Jacquet ME, Roche D, Barbe V, Castellanos M, Bouet JY, Cruveiller S, Médigue C, Blanco J, Clermont O, Denamur E, Branger C. 2014. Characterization of a P1-like bacteriophage carrying an SHV-2 extended-spectrum β-lactamase from an *Escherichia coli* strain. Antimicrob Agents Chemother 58:6550–6557. doi:10.1128/AAC.03183-14. 25136025 PMC4249366

[28] Venturini C, Zingali T, Wyrsch ER, Bowring B, Iredell J, Partridge SR, Djordjevic SP. 2019. Diversity of P1 phage-like elements in multidrug resistant *Escherichia coli*. Sci Rep 9:18861. doi:10.1038/s41598-019-54895-4. 31827120 PMC6906374

[29] Feldgarden M, Brover V, Gonzalez-Escalona N, Frye JG, Haendiges J, Haft DH, Hoffmann M, Pettengill JB, Prasad AB, Tillman GE, Tyson GH, Klimke W. 2021. AMRFinderPlus and the Reference Gene Catalog facilitate examination of the genomic links among antimicrobial resistance, stress response, and virulence. Sci Rep 11:12728. doi:10.1038/s41598-021-91456-0. 34135355 PMC8208984

[30] Murray CJ, Ikuta KS, Sharara F, Swetschinski L, Aguilar GR, Gray A, Han C, Bisignano C, Rao P, Wool E, Johnson SC, Browne AJ, Chipeta MG, Fell F, Hackett S, Haines-Woodhouse G, Hamadani BHK, Kumaran EAP, McManigal B, Agarwal R, Akech S, Albertson S, Amuasi J, Andrews J, Aravkin A, Ashley E, Bailey F, Baker S, Basnyat B, Bekker A, Bender R, Bethou A, Bielicki J, Boonkasidecha S, Bukosia J, Carvalheiro C, Castañeda-Orjuela C, Chansamouth V, Chaurasia S, Chiurchiù S, Chowdhury F, Cook AJ, Cooper B, Cressey TR, Criollo-Mora E, Cunningham M, Darboe S, Day NPJ, Luca MD, Dokova K, et al. 2022. Global burden of bacterial antimicrobial resistance in 2019: a systematic analysis. Lancet 399:629–655. doi:10.1016/S0140-6736(21)02724-0. 35065702 PMC8841637

[31] Gautreau G, Bazin A, Gachet M, Planel R, Burlot L, Dubois M, Perrin A, Médigue C, Calteau A, Cruveiller S, Matias C, Ambroise C, Rocha EPC, Vallenet D. 2020. PPanGGOLiN: depicting microbial diversity via a partitioned pangenome graph. PLoS Comput Biol 16:e1007732. doi:10.1371/journal.pcbi.1007732. 32191703 PMC7108747

[32] Ploy M-C, Berendonk TU. 2022. Editorial overview: AMR in the environment — too complex for surveillance? Curr Opin Microbiol 65:xi–xiii. doi:10.1016/j.mib.2021.10.015. 34774437

[33] Varani A, He S, Siguier P, Ross K, Chandler M. 2021. The IS6 family, a clinically important group of insertion sequences including IS26. Mob DNA 12:11. doi:10.1186/s13100-021-00239-x. 33757578 PMC7986276

[34] Gillings MR. 2014. Integrons: past, present, and future. Microbiol Mol Biol Rev 78:257–277. doi:10.1128/MMBR.00056-13. 24847022 PMC4054258

[35] Néron B, Littner E, Haudiquet M, Perrin A, Cury J, Rocha E. 2022. IntegronFinder 2.0: identification and analysis of integrons across bacteria, with a focus on antibiotic resistance in *Klebsiella*. Microorganisms 10:700. doi:10.3390/microorganisms10040700. 35456751 PMC9024848

[36] Refardt D. 2011. Within-host competition determines reproductive success of temperate bacteriophages. ISME J 5:1451–1460. doi:10.1038/ismej.2011.30. 21412345 PMC3160688

[37] Dowah ASA, Clokie MRJ. 2018. Review of the nature, diversity and structure of bacteriophage receptor binding proteins that target Gram-positive bacteria. Biophys Rev 10:535–542. doi:10.1007/s12551-017-0382-3. 29299830 PMC5899739

[38] Bernheim A, Sorek R. 2020. The pan-immune system of bacteria: antiviral defence as a community resource. 2. Nat Rev Microbiol 18:113–119. doi:10.1038/s41579-019-0278-2. 31695182

[39] Bondy-Denomy J, Qian J, Westra ER, Buckling A, Guttman DS, Davidson AR, Maxwell KL. 2016. Prophages mediate defense against phage infection through diverse mechanisms. ISME J 10:2854–2866. doi:10.1038/ismej.2016.79. 27258950 PMC5148200

[40] de Sousa JAM, Buffet A, Haudiquet M, Rocha EPC, Rendueles O. 2020. Modular prophage interactions driven by capsule serotype select for capsule loss under phage predation. ISME J 14:2980–2996. doi:10.1038/s41396-020-0726-z. 32732904 PMC7784688

[41] Piel D, Bruto M, Labreuche Y, Blanquart F, Chenivesse S, Lepanse S, James A, Barcia-Cruz R, Dubert J, Petton B, Lieberman E, Wegner KM, Hussain FA, Kauffman KM, Polz MF, Bikard D, Gandon S, Roux FL. 2021. Genetic determinism of phage-bacteria coevolution in natural populations. bioRxiv doi:10.1101/2021.05.05.442762.

[42] Bobay L-M, Touchon M, Rocha EPC. 2014. Pervasive domestication of defective prophages by bacteria. Proc Natl Acad Sci USA 111:12127–12132. doi:10.1073/pnas.1405336111. 25092302 PMC4143005

[43] Murooka Y, Harada T. 1979. Expansion of the host range of coliphage P1 and gene transfer from enteric bacteria to other gram-negative bacteria. Appl Environ Microbiol 38:754–757. doi:10.1128/aem.38.4.754-757.1979. 395900 PMC243574

[44] Iida S, Meyer J, Kennedy KE, Arber W. 1982. A site-specific, conservative recombination system carried by bacteriophage P1. Mapping the recombinase gene cin and the cross-over sites cix for the inversion of the C segment. EMBO J 1:1445–1453. doi:10.1002/j.1460-2075.1982.tb01336.x. 6327269 PMC553230

[45] Colavecchio A, Jeukens J, Freschi L, Edmond Rheault J-G, Kukavica-Ibrulj I, Levesque RC, LeJeune J, Goodridge L. 2017. Complete genome sequences of two phage-like plasmids carrying the CTX-M-15 extended-spectrum β-lactamase gene. Genome Announc 5:e00102-17. doi:10.1128/genomeA.00102-17. 28495759 PMC5427194

[46] Nordmann P, Dortet L, Poirel L. 2012. Carbapenem resistance in *Enterobacteriaceae*: here is the storm!. Trends Mol Med 18:263–272. doi:10.1016/j.molmed.2012.03.003. 22480775

[47] Liu Y-Y, Wang Y, Walsh TR, Yi L-X, Zhang R, Spencer J, Doi Y, Tian G, Dong B, Huang X, Yu L-F, Gu D, Ren H, Chen X, Lv L, He D, Zhou H, Liang Z, Liu J-H, Shen J. 2016. Emergence of plasmid-mediated colistin resistance mechanism MCR-1 in animals and human beings in China: a microbiological and molecular biological study. Lancet Infect Dis 16:161–168. doi:10.1016/S1473-3099(15)00424-7. 26603172

[48] Galimand M, Courvalin P, Lambert T. 2012. RmtF, a new member of the aminoglycoside resistance 16S rRNA N7 G1405 methyltransferase family. Antimicrob Agents Chemother 56:3960–3962. doi:10.1128/AAC.00660-12. 22547620 PMC3393463

[49] Paepe MD, Taddei F. 2006. Viruses’ life history: towards a mechanistic basis of a trade-off between survival and reproduction among phages. PLoS Biol 4:e193. doi:10.1371/journal.pbio.0040193. 16756387 PMC1475768

[50] Zhang X, McDaniel AD, Wolf LE, Keusch GT, Waldor MK, Acheson DWK. 2000. Quinolone antibiotics induce Shiga toxin-encoding bacteriophages, toxin production, and death in mice. J Infect Dis 181:664–670. doi:10.1086/315239. 10669353

[51] Leclercq S, Cordaux R. 2011. Do phages efficiently shuttle transposable elements among prokaryotes? Evolution 65:3327–3331. doi:10.1111/j.1558-5646.2011.01395.x. 22023596

[52] Stalder T, Barraud O, Casellas M, Dagot C, Ploy M-C. 2012. Integron involvement in environmental spread of antibiotic resistance. Front Microbiol 3. doi:10.3389/fmicb.2012.00119. PMC3321497 22509175

[53] Moura de Sousa JA, Pfeifer E, Touchon M, Rocha EPC. 2021. Causes and consequences of bacteriophage diversification via genetic exchanges across lifestyles and bacterial taxa. Mol Biol Evol 38:2497–2512. doi:10.1093/molbev/msab044. 33570565 PMC8136500

[54] Latino L, Midoux C, Hauck Y, Vergnaud G, Pourcel C. 2016. Pseudolysogeny and sequential mutations build multiresistance to virulent bacteriophages in *Pseudomonas aeruginosa*. Microbiology (Reading) 162:748–763. doi:10.1099/mic.0.000263. 26921273

[55] Pruitt KD, Tatusova T, Maglott DR. 2007. NCBI reference sequences (RefSeq): a curated non-redundant sequence database of genomes, transcripts and proteins. Nucleic Acids Res 35:D61–D65. doi:10.1093/nar/gkl842. 17130148 PMC1716718

[56] Steinegger M, Söding J. 2017. MMseqs2 enables sensitive protein sequence searching for the analysis of massive data sets. Nat Biotechnol 35:1026–1028. doi:10.1038/nbt.3988. 29035372

[57] Grazziotin AL, Koonin EV, Kristensen DM. 2017. Prokaryotic virus orthologous groups (pVOGs): a resource for comparative genomics and protein family annotation. Nucleic Acids Res 45:D491–D498. doi:10.1093/nar/gkw975. 27789703 PMC5210652

[58] Mistry J, Chuguransky S, Williams L, Qureshi M, Salazar GA, Sonnhammer ELL, Tosatto SCE, Paladin L, Raj S, Richardson LJ, Finn RD, Bateman A. 2021. Pfam: the protein families database in 2021. Nucleic Acids Res 49:D412–D419. doi:10.1093/nar/gkaa913. 33125078 PMC7779014

[59] Haft DH, Loftus BJ, Richardson DL, Yang F, Eisen JA, Paulsen IT, White O. 2001. TIGRFAMs: a protein family resource for the functional identification of proteins. Nucleic Acids Res 29:41–43. doi:10.1093/nar/29.1.41. 11125044 PMC29844

[60] Eddy SR. 2011. Accelerated profile HMM searches. PLoS Comput Biol 7:e1002195. doi:10.1371/journal.pcbi.1002195. 22039361 PMC3197634

[61] Touchon M, Bernheim A, Rocha EP. 2016. Genetic and life-history traits associated with the distribution of prophages in bacteria. 11. ISME J 10:2744–2754. doi:10.1038/ismej.2016.47. 27015004 PMC5113838

[62] Harrison PW, Lower RPJ, Kim NKD, Young JPW. 2010. Introducing the bacterial ‘chromid’: not a chromosome, not a plasmid. Trends Microbiol 18:141–148. doi:10.1016/j.tim.2009.12.010. 20080407

[63] Cury J, Touchon M, Rocha EPC. 2017. Integrative and conjugative elements and their hosts: composition, distribution and organization. Nucleic Acids Res 45:8943–8956. doi:10.1093/nar/gkx607. 28911112 PMC5587801

[64] Alcock BP, Raphenya AR, Lau TTY, Tsang KK, Bouchard M, Edalatmand A, Huynh W, Nguyen A-LV, Cheng AA, Liu S, Min SY, Miroshnichenko A, Tran H-K, Werfalli RE, Nasir JA, Oloni M, Speicher DJ, Florescu A, Singh B, Faltyn M, Hernandez-Koutoucheva A, Sharma AN, Bordeleau E, Pawlowski AC, Zubyk HL, Dooley D, Griffiths E, Maguire F, Winsor GL, Beiko RG, Brinkman FSL, Hsiao WWL, Domselaar GV, McArthur AG. 2020. CARD 2020: antibiotic resistome surveillance with the comprehensive antibiotic resistance database. Nucleic Acids Res 48:D517–D525. 31665441 10.1093/nar/gkz935 PMC7145624

[65] Bortolaia V, Kaas RS, Ruppe E, Roberts MC, Schwarz S, Cattoir V, Philippon A, Allesoe RL, Rebelo AR, Florensa AF, Fagelhauer L, Chakraborty T, Neumann B, Werner G, Bender JK, Stingl K, Nguyen M, Coppens J, Xavier BB, Malhotra-Kumar S, Westh H, Pinholt M, Anjum MF, Duggett NA, Kempf I, Nykäsenoja S, Olkkola S, Wieczorek K, Amaro A, Clemente L, Mossong J, Losch S, Ragimbeau C, Lund O, Aarestrup FM. 2020. ResFinder 4.0 for predictions of phenotypes from genotypes. J Antimicrob Chemother 75:3491–3500. doi:10.1093/jac/dkaa345. 32780112 PMC7662176

[66] Gupta SK, Padmanabhan BR, Diene SM, Lopez-Rojas R, Kempf M, Landraud L, Rolain J-M. 2014. ARG-ANNOT, a new bioinformatic tool to discover antibiotic resistance genes in bacterial genomes. Antimicrob Agents Chemother 58:212–220. doi:10.1128/AAC.01310-13. 24145532 PMC3910750

[67] Camacho C, Coulouris G, Avagyan V, Ma N, Papadopoulos J, Bealer K, Madden TL. 2009. BLAST+: architecture and applications. BMC Bioinform 10:421. doi:10.1186/1471-2105-10-421. PMC2803857 20003500

[68] Xie Z, Tang H. 2017. ISEScan: automated identification of insertion sequence elements in prokaryotic genomes. Bioinformatics 33:3340–3347. doi:10.1093/bioinformatics/btx433. 29077810

[69] Hyatt D, Chen G-L, LoCascio PF, Land ML, Larimer FW, Hauser LJ. 2010. Prodigal: prokaryotic gene recognition and translation initiation site identification. BMC Bioinform 11:119. doi:10.1186/1471-2105-11-119. PMC2848648 20211023

[70] Pitcher Dg, Saunders Na, Owen RJ. 1989. Rapid extraction of bacterial genomic DNA with guanidium thiocyanate. Lett Appl Microbiol 8:151–156. doi:10.1111/j.1472-765X.1989.tb00262.x.

[71] Kolmogorov M, Yuan J, Lin Y, Pevzner PA. 2019. Assembly of long, error-prone reads using repeat graphs. Nat Biotechnol 37:540–546. doi:10.1038/s41587-019-0072-8. 30936562

[72] Jakočiūnė D, Moodley A. 2018. A rapid bacteriophage DNA extraction method. MPs 1:27. doi:10.3390/mps1030027. PMC6481073 31164569

[73] Wick RR, Judd LM, Gorrie CL, Holt KE. 2017. Unicycler: resolving bacterial genome assemblies from short and long sequencing reads. PLoS Comput Biol 13:e1005595. doi:10.1371/journal.pcbi.1005595. 28594827 PMC5481147

[74] Langmead B, Salzberg SL. 2012. Fast gapped-read alignment with Bowtie 2. Nat Methods 9:357–359. doi:10.1038/nmeth.1923. 22388286 PMC3322381

[75] Li H, Handsaker B, Wysoker A, Fennell T, Ruan J, Homer N, Marth G, Abecasis G, Durbin R, 1000 Genome Project Data Processing Subgroup. 2009. The Sequence Alignment/Map format and SAMtools. Bioinformatics 25:2078–2079. doi:10.1093/bioinformatics/btp352. 19505943 PMC2723002

[76] Quinlan AR, Hall IM. 2010. BEDTools: a flexible suite of utilities for comparing genomic features. Bioinformatics 26:841–842. doi:10.1093/bioinformatics/btq033. 20110278 PMC2832824

[77] Bonnin RA, Emeraud C, Jousset AB, Naas T, Dortet L. 2022. Comparison of disk diffusion, MIC test strip and broth microdilution methods for cefiderocol susceptibility testing on carbapenem-resistant enterobacterales. Clin Microbiol Infect S1198-743X(22)00221-X. 10.1016/j.cmi.2022.04.013 35533970

[78] Łobocka MB, Rose DJ, Plunkett G, Rusin M, Samojedny A, Lehnherr H, Yarmolinsky MB, Blattner FR. 2004. Genome of bacteriophage P1. J Bacteriol 186:7032–7068. doi:10.1128/JB.186.21.7032-7068.2004. 15489417 PMC523184

