# Supplementary material for: Phage-Plasmids Spread Antibiotic Resistance Genes through Infection and Lysogenic Conversion
Source: mBio. 2022 Sep 26;13(5):e01851-22. doi: 10.1128/mbio.01851-22 (PMC9600943; doi:10.1128/mbio.01851-22)

A.

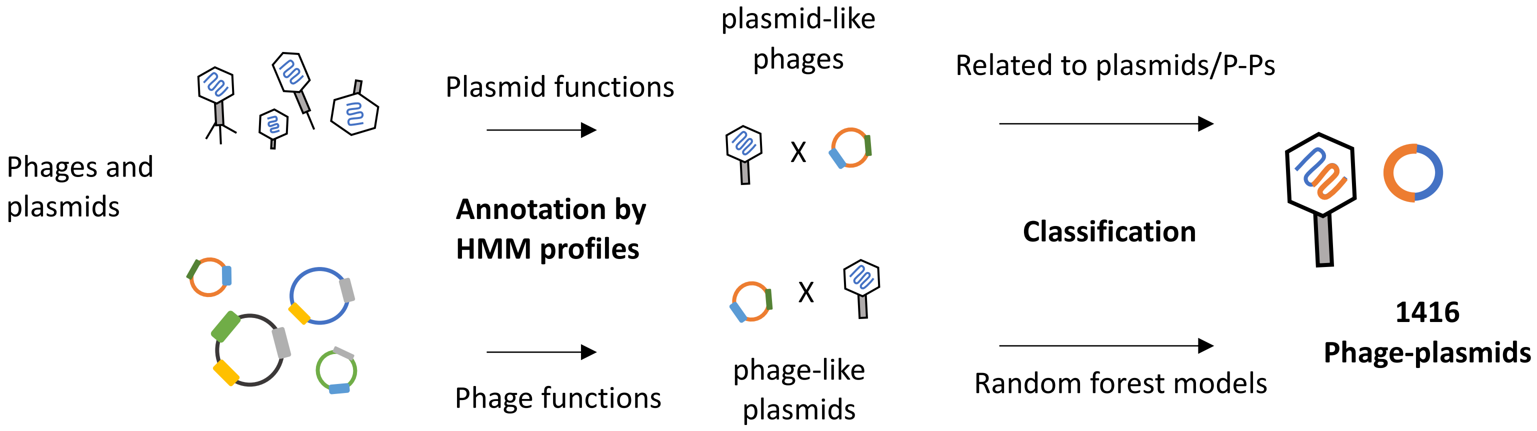

B.

Database version	AB-group	SSU5-supergroup (pCAV, pKpn, pMT1, pSLy3, SSU5_pHMC2)	P1-group	N15-group
Mai 2019	24	145 (8+38+35+29+35)	64	42
March 2021	40	268 (14+94+43+62+55)	149	71

C.

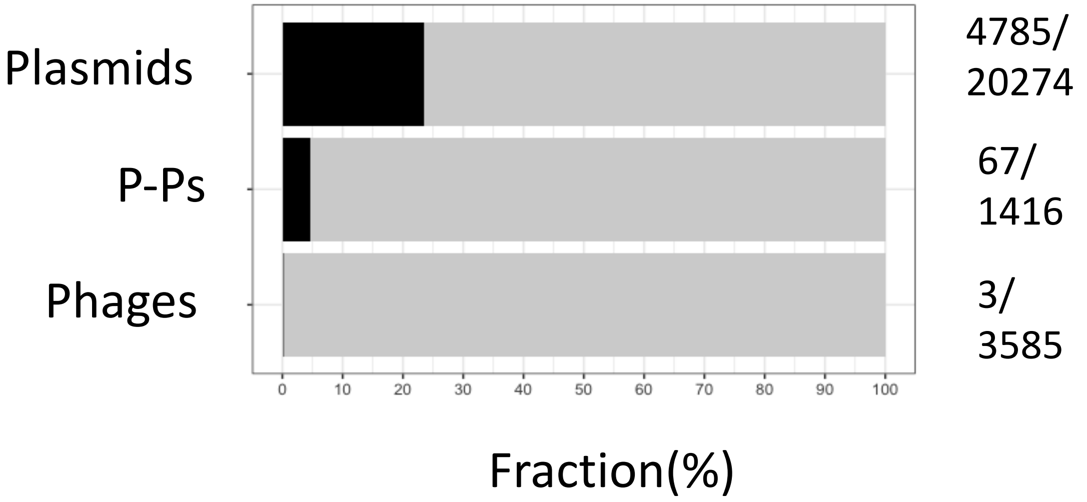

Supplement: FIG S1 [file mbio.01851-22-s0006.pdf]
