# Supplementary figures and images for: Phage-Plasmids Spread Antibiotic Resistance Genes through Infection and Lysogenic Conversion
Source: mBio. 2022 Sep 26;13(5):e01851-22. doi: 10.1128/mbio.01851-22 (PMC9600943; doi:10.1128/mbio.01851-22)

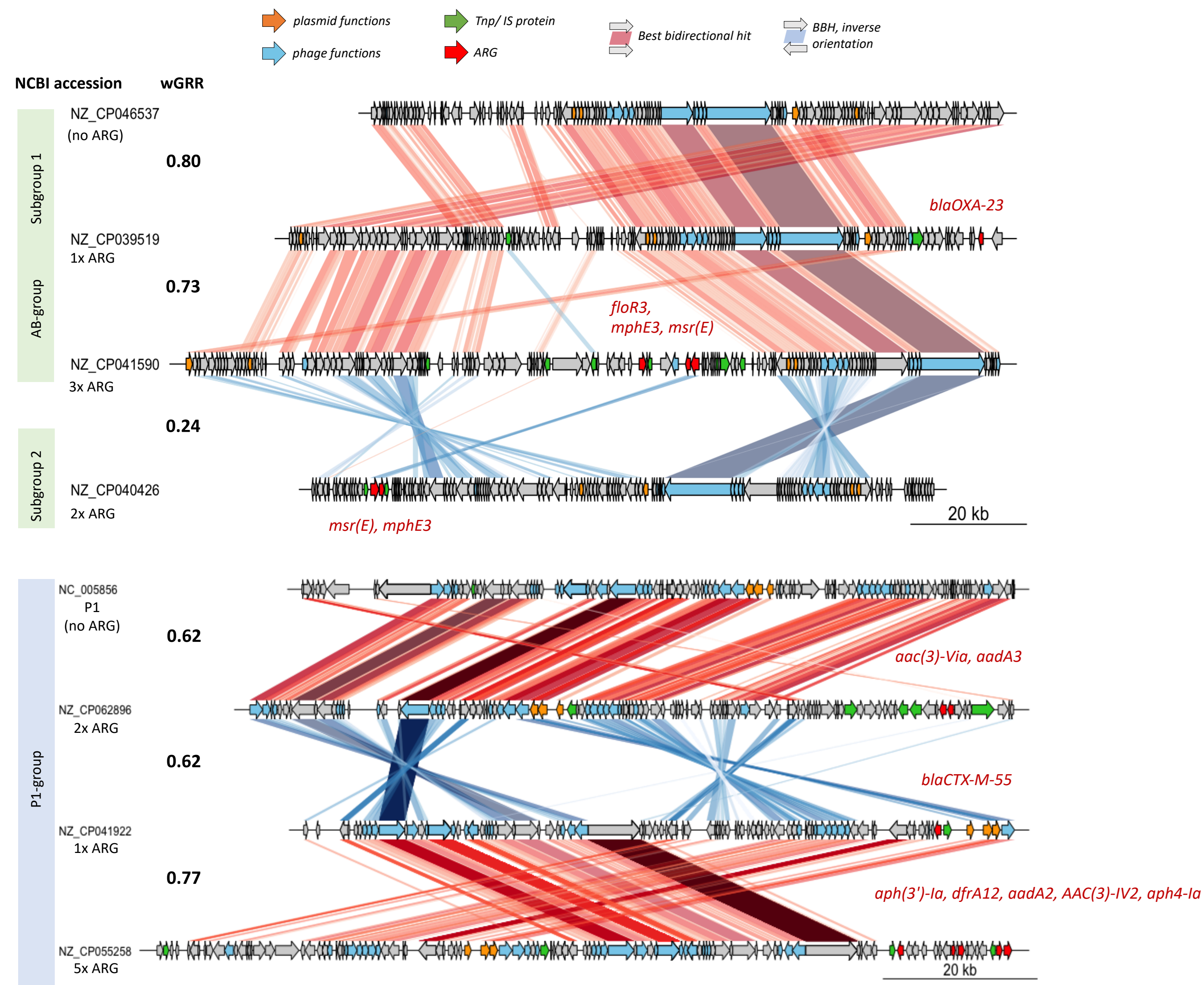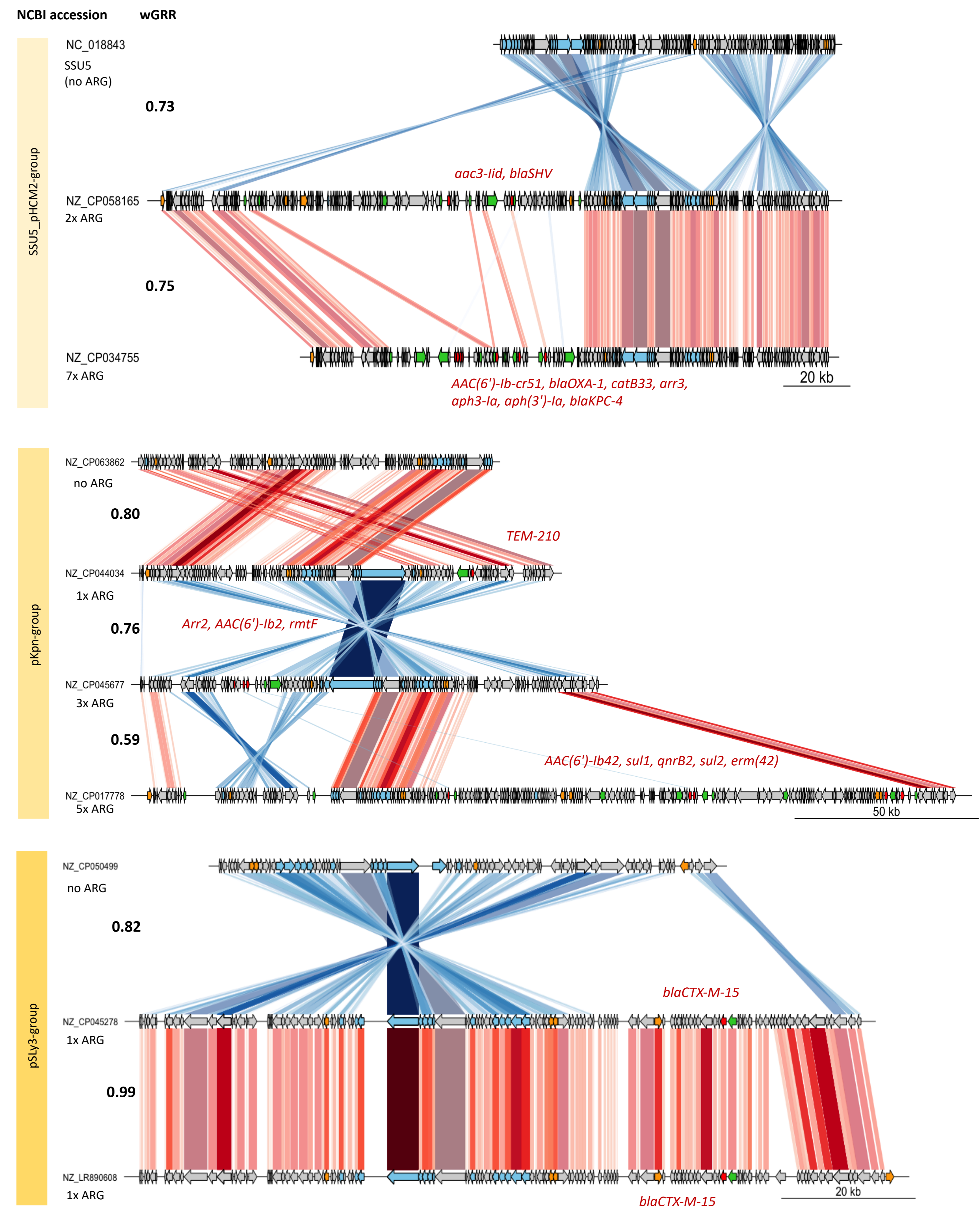

Supplement: FIG S2 [file mbio.01851-22-s0007.pdf]

A.

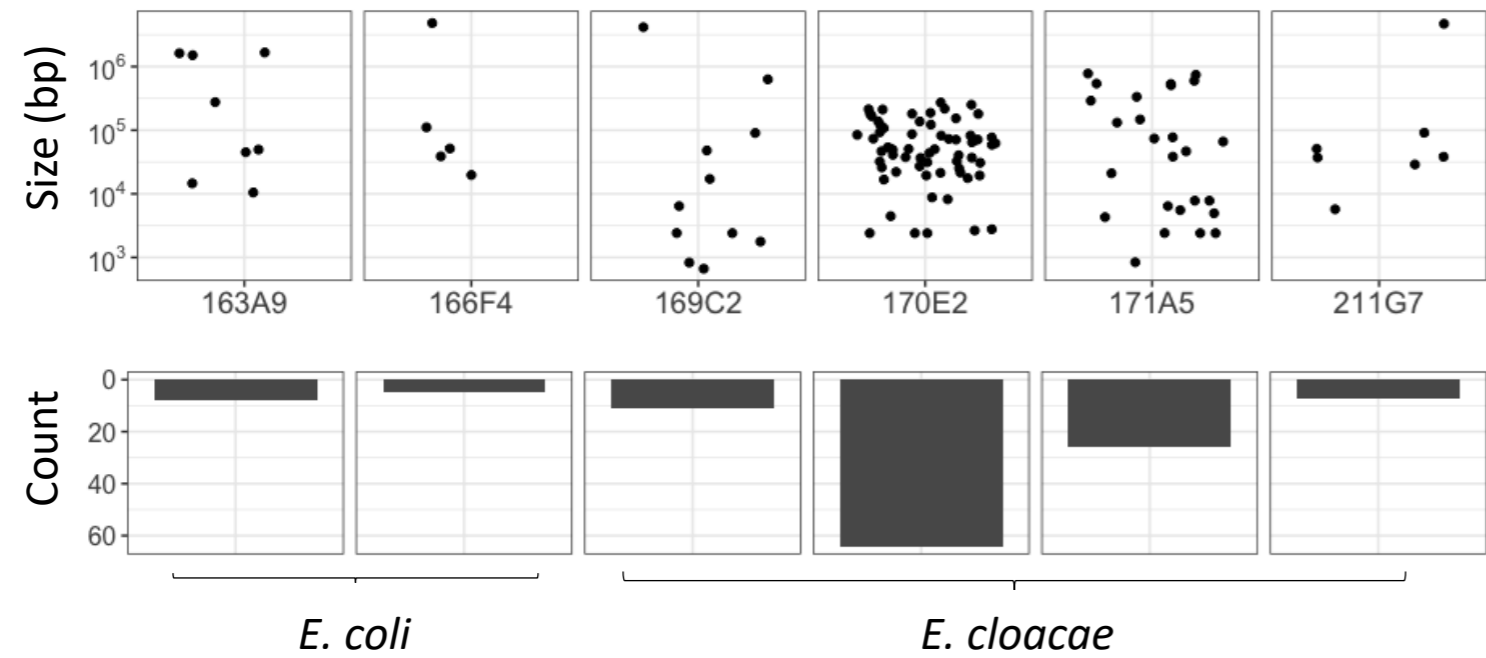

B.

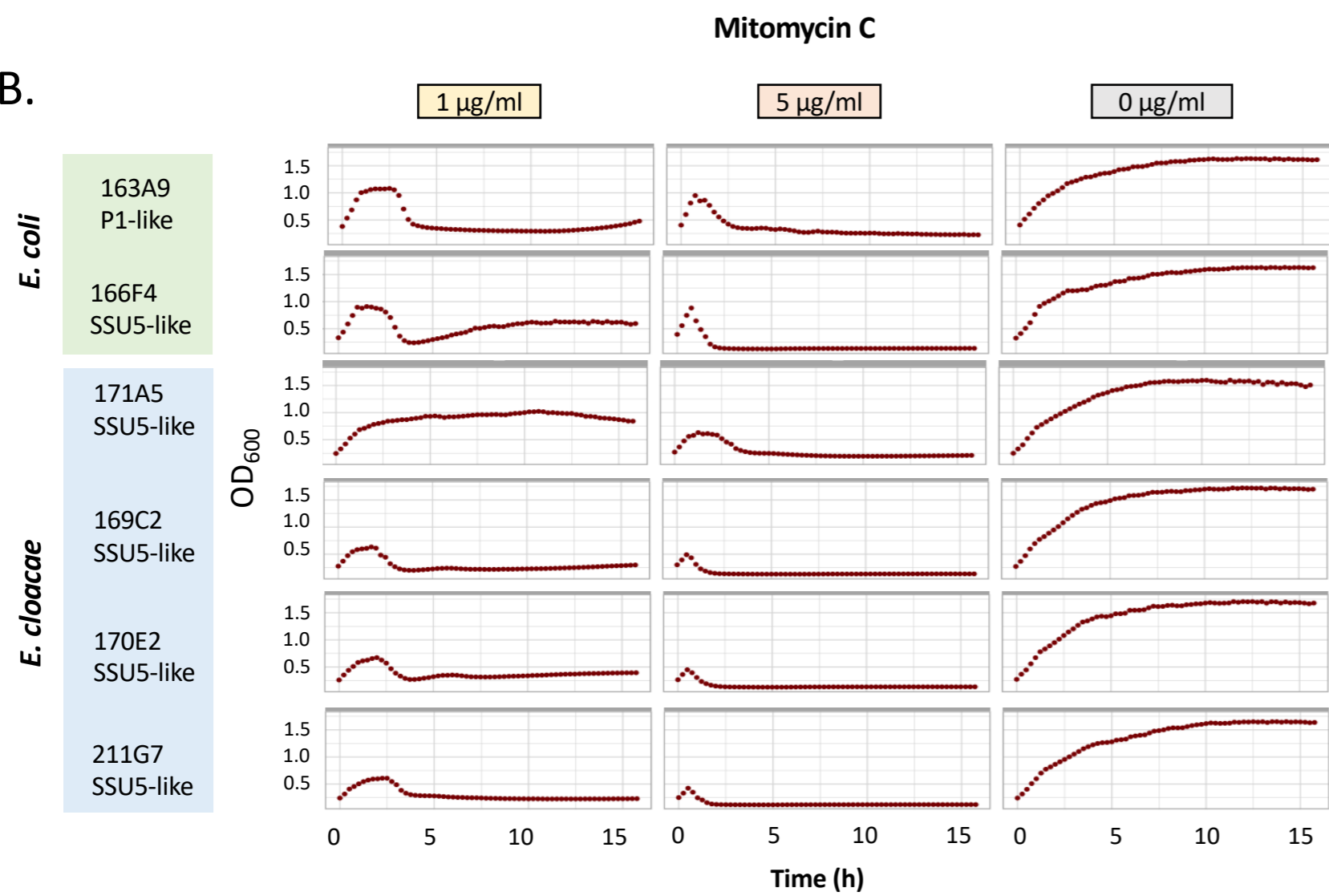

C.

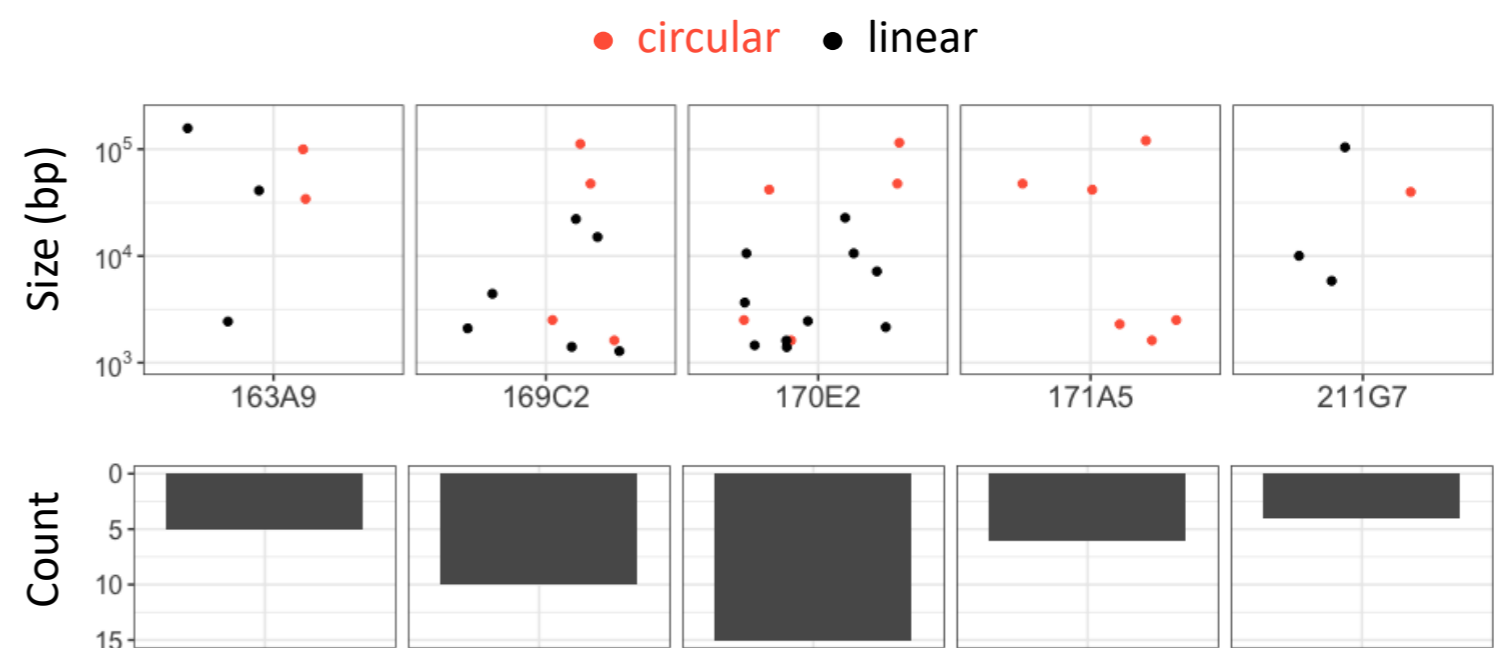

D.

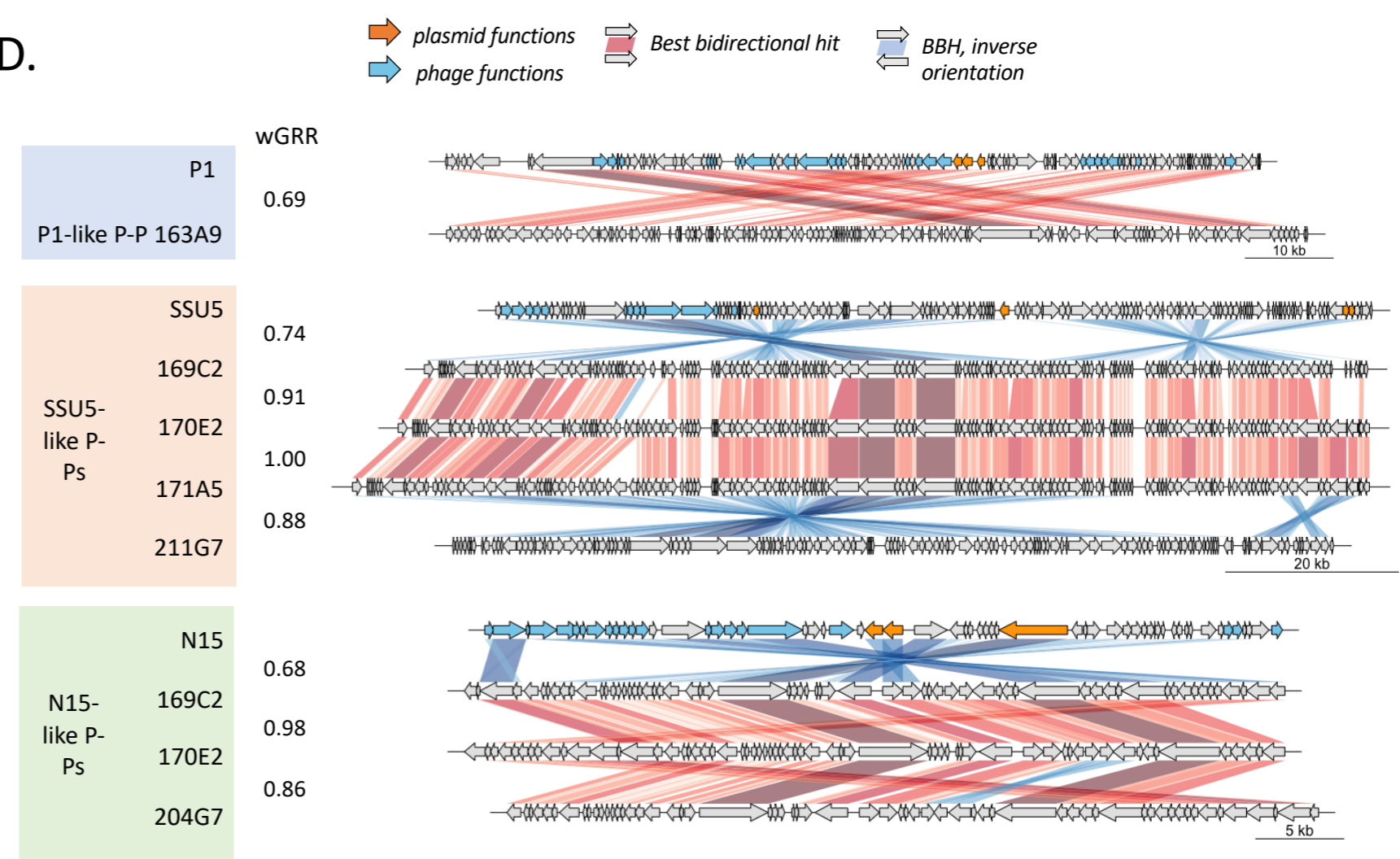

Supplement: FIG S3 [file mbio.01851-22-s0008.pdf]
