# Supplementary material for: Phage-Plasmids Spread Antibiotic Resistance Genes through Infection and Lysogenic Conversion
Source: mBio. 2022 Sep 26;13(5):e01851-22. doi: 10.1128/mbio.01851-22 (PMC9600943; doi:10.1128/mbio.01851-22)
Supplement: FIG S4 [file mbio.01851-22-s0009.pdf]

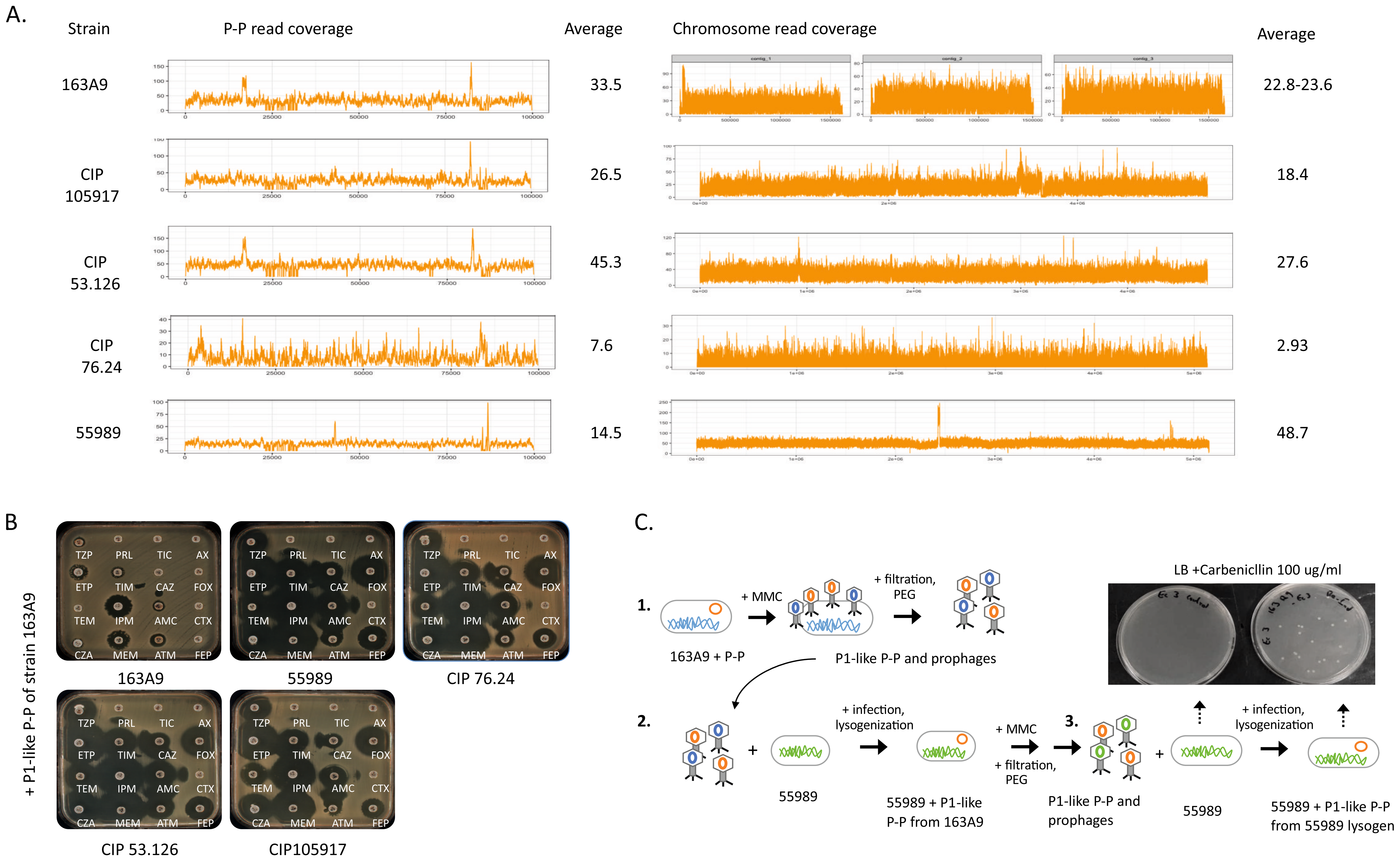

TZP: piperacillin/tazobactam
PRL: piperacillin
TIC: ticarcillin
AX: Amoxicillin
ETP: ertapenem
TIM: Ticarcillin/clavulanate
CAZ: ceftazidime
FOX: cefoxitine
TEM: temocillin
IPM: imipenem
AMC: Amoxicillin/clavulanate
CTX: cefotaxime
CZA:ceftazidime/avibactam
MEM: meropenem
ATM: aztreonam
FEP: cefepime
